# Applications of Artificial Intelligence Based on Medical Imaging in Glioma: Current State and Future Challenges

**DOI:** 10.3389/fonc.2022.892056

**Published:** 2022-07-27

**Authors:** Jiaona Xu, Yuting Meng, Kefan Qiu, Win Topatana, Shijie Li, Chao Wei, Tianwen Chen, Mingyu Chen, Zhongxiang Ding, Guozhong Niu

**Affiliations:** ^1^ Hangzhou First People’s Hospital, Zhejiang University School of Medicine, Hangzhou, China; ^2^ Department of General Surgery, Sir Run-Run Shaw Hospital, Zhejiang University School of Medicine, Hangzhou, China; ^3^ Department of Neurology, Affiliated Ningbo First Hospital, Ningbo, China; ^4^ Department of Neurology, Affiliated Hangzhou First People’s Hospital, Zhejiang University School of Medicine, Hangzhou, China; ^5^ Department of Radiology, Affiliated Hangzhou First People’s Hospital, Zhejiang University School of Medicine, Hangzhou, China

**Keywords:** artificial intelligence, medical imaging, neural tumors, glioma, radiomics, machine learning, deep learning

## Abstract

Glioma is one of the most fatal primary brain tumors, and it is well-known for its difficulty in diagnosis and management. Medical imaging techniques such as magnetic resonance imaging (MRI), positron emission tomography (PET), and spectral imaging can efficiently aid physicians in diagnosing, treating, and evaluating patients with gliomas. With the increasing clinical records and digital images, the application of artificial intelligence (AI) based on medical imaging has reduced the burden on physicians treating gliomas even further. This review will classify AI technologies and procedures used in medical imaging analysis. Additionally, we will discuss the applications of AI in glioma, including tumor segmentation and classification, prediction of genetic markers, and prediction of treatment response and prognosis, using MRI, PET, and spectral imaging. Despite the benefits of AI in clinical applications, several issues such as data management, incomprehension, safety, clinical efficacy evaluation, and ethical or legal considerations, remain to be solved. In the future, doctors and researchers should collaborate to solve these issues, with a particular emphasis on interdisciplinary teamwork.

## Introduction

Glioma is the most common histological type of primary central nervous system cancer, accounting for 81% of all malignant brain tumors (1). Astrocytomas, oligodendrogliomas, oligoastrocytomas, and ependymomas are all types of gliomas. The World Health Organization (WHO) defines gliomas into four categories; the first two grades and the last two grades are further classified as low-grade glioma (LGG) and high-grade glioma (HGG). The poor 5-year overall survival (OS) rate for WHO grade IV glioma patients are 6.8% (2, 3). Glioblastoma (GBM) is the most aggressive kind of grade IV astrocytoma, accounting for 45% of gliomas and the 5-year OS rate of GBM patients is 5%. Treatment for gliomas generally comprises surgical excision, radiation, and temozolomide chemotherapy. Previous randomized clinical studies indicated that the addition of tumor-treating fields to routine treatment increased life expectancy by 4 months (4, 5).

Glioma diagnosis and treatment mostly involve imaging, segmentation and localization, grading, pathology, gene acquisition, and post-treatment recurrence monitoring (6, 7). Tumor treatment and decision-making are difficult due to the heterogeneity of tumors. Therefore, the rise of artificial intelligence (AI) has significantly alleviated doctors’ loads (8, 9). AI enables physicians to examine therapeutically important material that is buried inside massive volumes of data (10). Precision medicine is based on artificial intelligence, a relatively new technique to diagnose and treat a disease that considers various factors such as genetics, environment, and lifestyle. Magnetic resonance imaging (MRI), positron emission tomography (PET), and spectral imaging of the brain all contain a wealth of structural and functional information that can be analyzed by AI algorithms for glioma patient management and decision-making (11). However, neurologists should be aware of its limitations, since the use of algorithms raises concerns regarding transparency, privacy, data encryption, and licensing (12). Additionally, doctors and scientists must bridge gaps in one another’s subject expertise (13).

The purpose of this review is to (1) provide an overview of AI technology and its applications in medical imaging analysis; (2) summarize the application and performance of AI-based on MRI, PET, and spectral images in glioma; and (3) discuss future challenges and directions for AI applications in the field of neural tumors.

## 1 Artificial Intelligence

**Figure 1:**
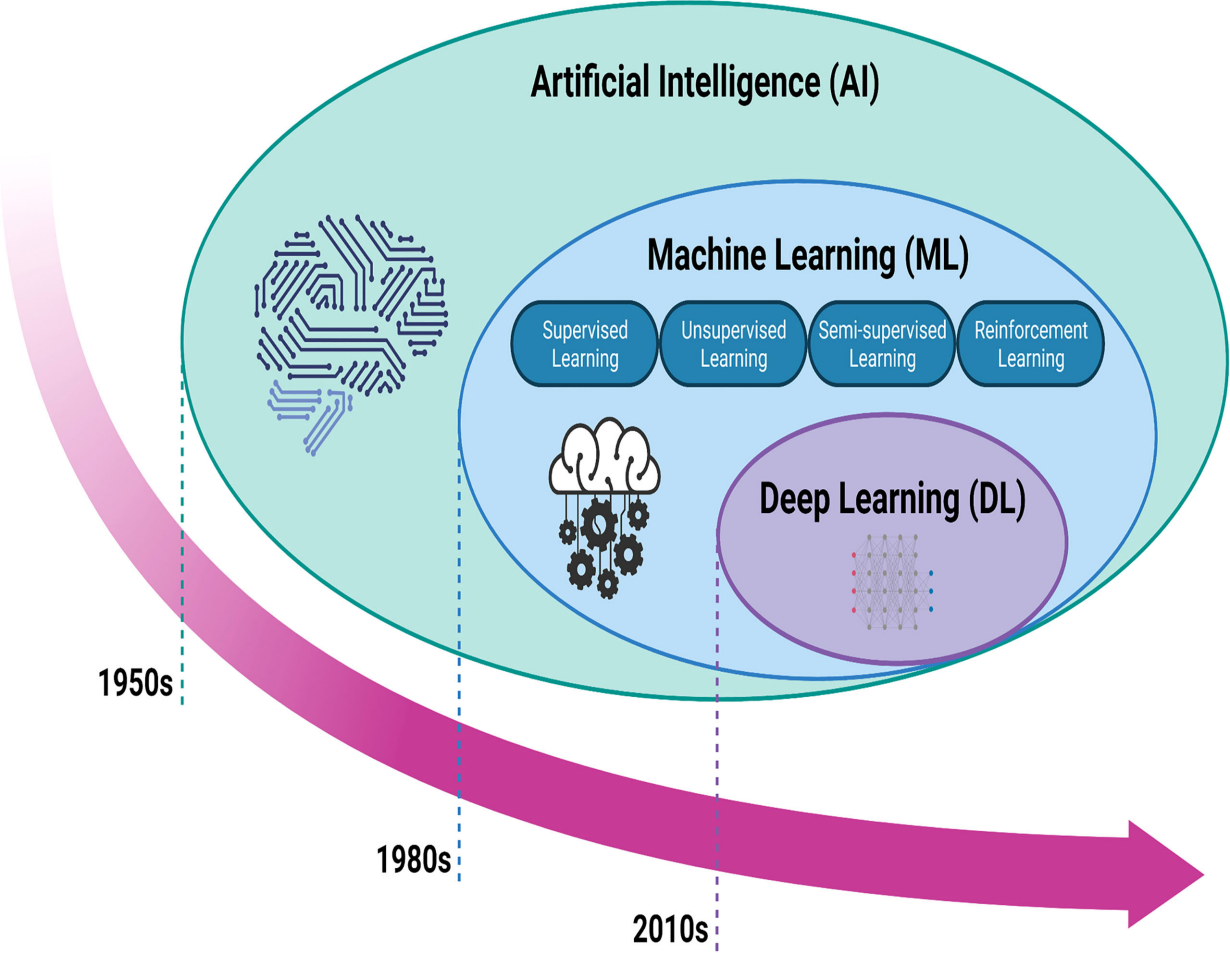
Artificial intelligence methods and timeline. Machine learning is a form of artificial intelligence that could be classified as supervised learning, unsupervisd learning, semisupervised learning, and reinforcement learning. Deep learning is a form of machine learning. AI: artificial intelligence; ML: machine learning; DL: deep learning.

AI broadly refers to the capacity of computers to emulate intelligent tasks, such as explicit rule-based systems and computer algorithms that do not require hard-coded rules (14). It was first proposed by an American computer scientist John McCarthy in 1956 (15). Subsequently, machine learning (ML), which falls under the umbrella of AI, has emerged and been applied in various fields. In the past decade, deep learning (DL), a new model of ML, has shown great potential for applications in a broader range of domains, leading to the third AI boom (16, 17) (**Figure 1**).

### 1.1 Machine Learning

ML is a subfield of AI that integrates algorithms and statistical models trained on sample data, allowing computers to learn from previously-stored “training” data without explicitly programming to anticipate new data points (18). ML can be classified as supervised, unsupervised, semi-supervised, and reinforcement learning. To forecast a regression or classification, supervised learning algorithms must be trained on a labeled dataset (19). The most often used supervised approaches include support vector machine (SVM), linear and logistic regression (LR), random forest (RF), decision trees, and Bayesian networks (20). Unsupervised learning algorithms can discover patterns by grouping unlabeled datasets or reducing data. Gaussian mixture modeling, affinity propagation, mean shift, K-mean clustering, and hierarchical clustering are all frequently used techniques. Semi-supervised learning is a technique that combines labeled and unlabeled data. It is a hybrid of supervised and unsupervised learning. Reinforcement learning is a machine learning-enhanced decision-making technique that develops algorithms for a specific task and learns from future errors and successes to reinforce learning (21).

Since the 1980s, ML has been used to create accurate predictions and classifications based on input data in different disciplines, including military research, life science, and clinical practice. This substantially contributed to the advancement of several fields and allowed AI development to again reach its pinnacle after the 1950s (22). However, the construction of every ML model requires intricate feature engineering, resulting in a convoluted workflow. Besides, the accuracy of ML is not satisfactory. Thus, the breadth and extent of ML applications are restricted, leading to the creation of DL (16, 23).

### 1.2 Deep Learning

Since the 2010s, the advent of DL has fundamentally altered the traditional model, in response to the past two AI booms (16). DL is a subset of ML that derives its technology from the artificial neural network (ANN) (24). In comparison to ML approaches, DL algorithms can identify underlying patterns in data without the requirement to extract individual features. The layer-by-layer updating of DL weights aids in the training of DL systems, while the ML weights are updated concurrently. The primary DL-based networks include a convolutional neural network (CNN), deep neural network (DNN), recurrent neural network (RNN), deep auto-encoder (DA), deep belief network (DBN), and deep Boltzmann machine (DBM). Apart from these, generative adversarial network (GAN) and variation auto-encoder (VAE) are two recent approaches for generative and unsupervised learning (25). CNN performs exceptionally well in picture identification; convolutional and pool layers extract obvious information, while fully connected layers conduct final classification. For comparison, CNN’s approach encompasses all current ImageNet Classification Challenge winners, with a category mistake rate of 3.6% to date. The development of deep learning models has increased the number of layer designs and the number of model architectures, loss functions, and optimizers available for network construction. Due to the unlimited range of potential computational networks, a significant number of designs have been suggested (for example, AlexNet, ZeNet, Visual Geometry Group (VGG) Net, GoogLeNet, ResNet, DenseNet, Super Resolution CNN, and U-net, among others). Transfer learning (TL) is a subset of DL, and because the weights generated from these networks trained on ImageNet can be applied to different tasks, such as medical pictures, this AI can significantly cut training time (26).

In conclusion, constructing DL models is more time-efficient, simpler, and can achieve greater performance compared to ML. Moreover, DL is readily adaptable to various domains and applications due to TL. Although the DL establishment procedure is straightforward, it requires huge data sets and expensive hardware equipment, therefore ML remains a viable option for smaller data sets (27). Additionally, on a task-specific basis, a tailored image-naive architecture may outperform a DL architecture (16).

## 2 AI in Medical Imaging

Over the past few decades, medical imaging techniques including computed tomography (CT), MRI, PET, and ultrasound have been used for early detection, diagnosis, and treatment of diseases (28). In clinical settings, the majority of medical image interpretation has been performed by human specialists such as radiologists and physicians (27). Due to the varying levels of expertise among physicians and the possible exhaustion of human specialists, clinical application of medical imaging has not yielded flawless outcomes. The situation has been improved by the use of AI (29). Following the progression of AI development, ML was initially applied to analyze medical imaging. However, developing ML models necessitates those medical specialists to give well-described regularities or patterns inherent in data, which is a challenge for non-experts in computer science to apply ML to investigate their studies (30). Consequently, DL has been developed and widely used in medical imaging in recent years. Instead of manually extracting features, DL can autonomously find meaningful and useful features in datasets allowing nonexperts in AI to effectively use DL for their research. Besides, with sufficient training data, DL models can achieve greater accuracy (31).

**Figure 2:**
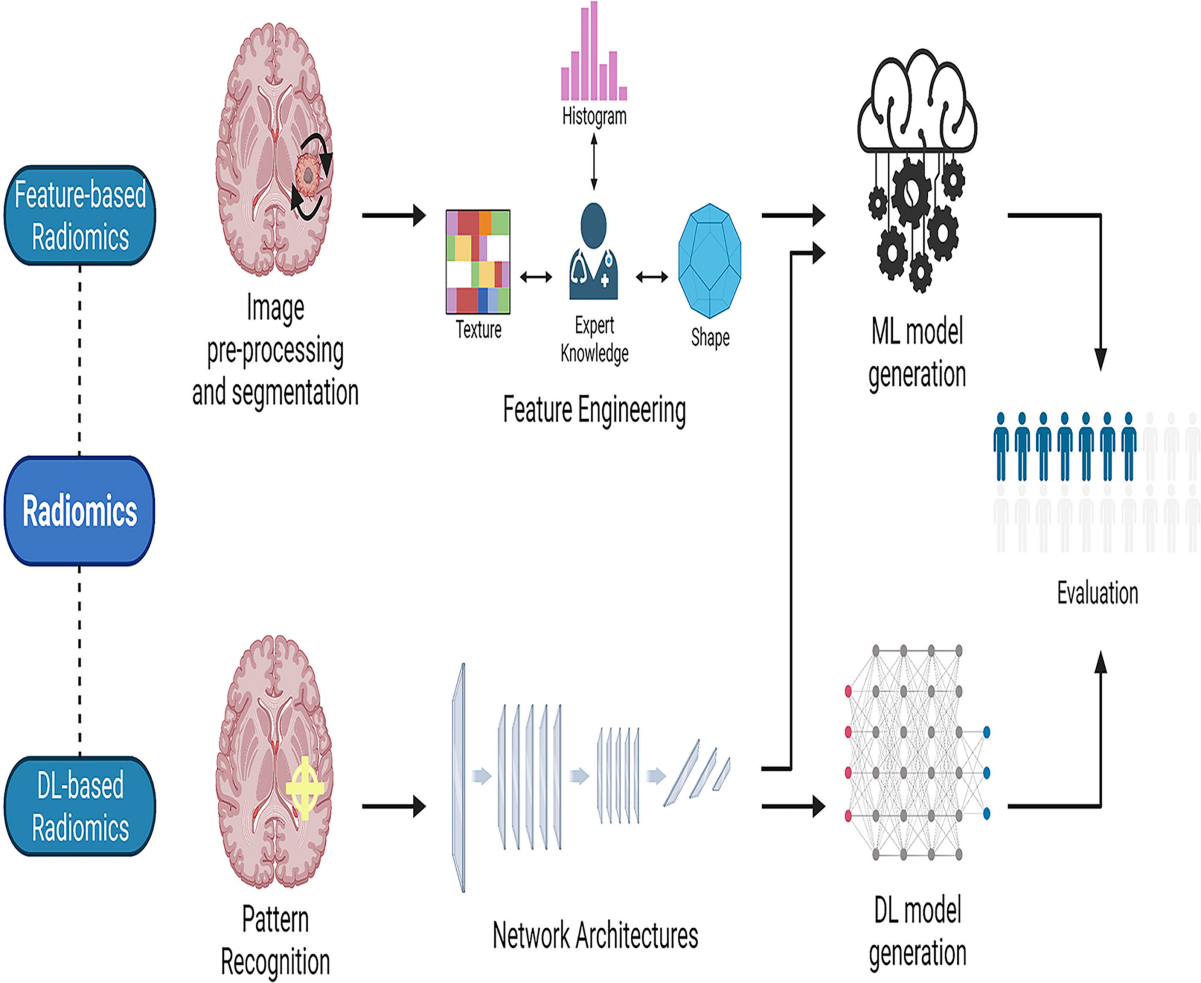
The workflow of radiomics. Radiomics may be divided into two categories: feature-based radiomics and deep learning-based radiomics. The workflow for feature-based radiomics begins with image preprocessing, tumor segmentation, feature extraction, and feature selection, and concludes with the construction and assessment of a mathematical model. In deep learning-based radiomics, different network architectures are used to find the most relevant features from the input data. Finally, the retrieved features can be processed further by the network for analysis and classification, or they can leave the network and used to generate models in a manner similar to the feature-based radiomics technique by employing different classifiers. ML, machine learning; DL, deep learning.

As different forms of AI techniques continue to be applied to medical imaging, radiomics has arisen. Radiomics is the application of computer image processing to transform region-of-interest (ROI) image data into mineable high-dimensional feature data. AI models are constructed based on the extracted feature data to make disease-related diagnoses and predictions (32). This AI-assisted technology is of great interest to doctors and is widely used in clinical research. Radiomics can elicit measurable objective data that has previously been unavailable and establish its relationship to underlying biological processes (33). Radiomics may be mainly classified into two types: feature-based and deep learning-based radiomics (34). The workflow for feature-based radiomics consists primarily of picture preprocessing, tumor segmentation, feature extraction, and feature selection, followed by the establishment and evaluation of a mathematical model (35). By utilizing various network topologies, deep learning-based radiomics procedures discover and detect classification-related patterns in picture data (36). The feature structure is then merged to form higher-level abstraction features. Finally, the retrieved features can be evaluated further by the network or subjected to a model-building process that is used in feature-based radiomics (**Figure 2**). To assess the AI technique, the model may be tested either internally (through cross-validation or bootstrapping) or externally (by supervised learning). After training and testing the model, it is desirable to apply it to a third dataset, referred known as the external validation dataset. External validation datasets serve as the gold standard for assessing the performance, robustness, and dependability of AI models. Statistical metrics like as accuracy, area under the receiver operating characteristic curve (AUC), sensitivity, specificity, positive/negative predictive values, and dice similarity coefficient (DSC) or dice score can be used to evaluate the effectiveness of AI systems (37).

## 3 Applications of AI-Based on Medical Imaging in Glioma

Neuroimaging techniques, such as contrast-enhanced CT, MRI, PET, and spectral imaging, have been widely applied for the detection, treatment, and prognostic prediction of glioma. However, the numerous amounts of data generated by these techniques and the heterogeneity of tumors are miserable for physicians. AI-based medical imaging could help to release physicians from these large amounts of data by integrating the similarity of these figures and providing directions. This section will mainly demonstrate the strengths and shortages of the application of AI-based MRI, PET, and spectral imaging in glioma.

### 3.1 Magnetic Resonance Imaging

MRI reflects the tumor pathophysiological environment at the voxel level by utilizing geometric, histogram, and texture analysis methods for quantification and prediction of image-based biomarkers *via* radiomics. Compared to biopsy, MRI is a non-invasive method, which could provide relatively comprehensive information on tumors. Whereas MRI can help to get rid of ionizing radiation and interference from bone artifacts when compared to contrast-enhanced CT. Besides, multiple sequences such as T1-weighted (T1) and T2-weighted (T2) MRI can be applied, which means more information can be obtained through MRI. Among these sequences, T1 images often depict the glioma boundaries, and fluid-attenuated inversion recovery (FLAIR) and T2 images more clearly depict the tumor core (38). The integrity of the blood-brain barrier (BBB) is disrupted in almost all high-grade gliomas, which means that the gadolinium-based contrast agents (GBCA) injected from the vein can successfully enter the extravascular extracellular space of the brain, manifesting as contrast-enhancing hyperintense regions on T1 sequences (39). Along with the T1 and T2 sequences, several other sequences have also been used to comprehensively evaluate the state of glioma (40). In detail, diffusion-weighted imaging (DWI), diffusion tensor imaging (DTI), and diffusional kurtosis imaging (DKI) can indicate changes in cell density, membrane permeability, and tissue microstructure; perfusion-weighted imaging (PWI) can detect changes in the microcirculation and cell proliferation (41); magnetic resonance spectroscopy (MRS) can reveal the metabolic status of malignancies directly, which is most closely connected to gene expression regulation, suggesting the combination of these two methods (42). Herein, we will discuss the application of AI-based MRI in glioma from the following four aspects: tumor segmentation and classification, molecular marker prediction, molecular marker prediction, and tumor cell analysis (**Supplementary Table 1**).

#### 3.1.1 Tumor Segmentation and Classification

Glioma is classified into four subtypes: enhanced area, non-enhanced region, necrosis area, and edema area. Several algorithms have been used to segment glioma. Among them, the outstanding performance of CNN has been well known in glioma segmentation, with an accuracy greater than 80-90%. Fu et al. (43) evaluated their multipath denseNet architecture based on 3D CNNs using the Brain Tumor Segmentation (BraTS) 2019 dataset and obtained a DSC of 0.922. Along with the CNN model mentioned above, other AI methodologies have also been applied in glioma segmentation. Another study combined Superpixel fuzzy clustering with the lattice Boltzmann technique can reach a disc of 0.93 (BraTS 2017) (44), demonstrating that the approach is resistant to noise, initialization, and strength inhomogeneity. Besides, Amin et al. (45) proposed a technique merging Local Binary Pattern and Gabor Wavelet Transform features, and generated dices of 0.96 (BraTS 2013), 0.98 (BraTS 2015), and 0.95 (local dataset). In summary, segmentation of glioma is a time-consuming and subjective task through the current manual ways. Through AI-based MRI, these shortages can be largely overcome, and subsequently, radiomics can be performed. Despite this, the large heterogeneity of HGG and the low proliferative state of LGG still bring a huge challenge to this task (71, 72). Besides, the various outcomes in the same datasets caused by different ML methodologies are a major concern for the application of ML in clinical. For instance, the results generated by a two-stage cascaded U-Net (73) and an RDAU-Net (74) using the BraTS 2019 training dataset which comprises 259 cases of HGG and 76 cases of LGG are various.

Additionally, the value of MRI in the grading and categorization of glioma has also been assessed according to its pathophysiology, molecular composition, and transcriptional activity. DL-based MRI, particularly CNN, performed well in a study of glioma classification and grading. For instance, Quon et al. created a modified ResNeXt-50-32x4d architecture to detect and classify gliomas into distinct pathological sub-types using T2 images (46), and this model demonstrated an AUC of 99% for tumor detection and 92% for glioma classification. In 2020, Basha et al. proposed a novel Harris Hawks optimization algorithm for evolving CNN architecture and investigated the classification and grading of brain tumors using two datasets; the former contains 8.000 brain tumors with four grades and 8.000 healthy MRI images, while the latter contains 4.908 MRI images with glioma, pituitary, and meningioma; the accuracy was greater than 95% in all experiments. Luo and colleagues (47) examined the utility of high-throughput network characteristics derived from the 3D U-net for histological and molecular subtype prediction in three cohorts of 655 glioma patients using conventional MRI. For histological diagnosis and molecular subtyping, the novel picture signature-based radiomics model achieved accuracies of 89.8% and 86.1% in the cross-validation cohort and 83.9% and 80.4% in the independent testing cohort. Overall, these studies indicated the high accuracy generated by DL in the grading of glioma.

Besides DL, other AI technologies also performed well in glioma classification. For example, Le et al. (48) identified transcriptome subgroups in GBM patients using conventional MRI in two cohorts of 120 patients. Model generation was performed using an eXtreme Gradient Boosting (XGBoost) machine classifier, and the model was constructed using 13 radiomics features selected from 704 handcrafted radiomics features achieved 70.9%, 73.3%, 88.4%, and 88.4% accuracy in predicting classical, mesenchymal, neural, and proneural subtypes, respectively. Lu and co-workers (49) achieved an accuracy of 81.8% after fivefold cross-validation using an SVM classifier based on radiomics features from multimodal MRI in 456 glioma patients for the classification of five molecular subtypes; this accuracy was increased to 89.2% when combined with histological diagnosis and MR radiomics.

In general, many AI systems can accurately detect and grade gliomas using picture data. However, because various studies use different data and defining criteria, it’s impossible to compare them, and it’s unclear which algorithm is the most effective.

#### 3.1.2 Molecular Marker Prediction

WHO included molecular and histological characteristics in the classification of brain cancers for the first time in 2016, and in 2021, WHO made significant revisions to the categorization of tumors, emphasizing the importance of molecular detection (75). The updated WHO 2016 classification of central nervous system malignancies stresses the prognostic significance of molecular characteristics such as the isocitrate dehydrogenase (IDH) genotype or the 1p/19q chromosomal arm heterozygous deletion (3). 2021 WHO classification approves methylome classifiers for a variety of CNS tumor types and subtypes and promoter methylation of O6-Methylguanine-DNA methyltransferase (MGMT) is related to an improved response to temozolomide therapy and a longer OS. Numerous studies have also demonstrated the predictive abilities of certain molecules. For example, research suggests that grade II or III IDH wild-type astrocytomas may harbor chromosomal +7/-10, epidermal growth factor receptor (EGFR) amplification, and/or telomerase reverse transcriptase promoter (TERT) alterations, with the same prognosis as GBMs (76). The detection of homozygous cyclin-dependent kinase inhibitor (CDKN) 2A/B deletion is critical for properly diagnosing and prognosing patients with diffuse astrocytomas caused by IDH mutations. In diffuse astrocytomas, IDH mutations are related to alpha thalassemia/intellectual disability syndrome X-linked (ATRX) and tumor protein 53 (TP53) functional loss mutations (77). ATRX mutations are mutually exclusive with co-deletion of 1p/19q and are associated with oligodendrocytes (78). TERT and ATRX are telomere maintenance proteins (79, 80).

Recent years have seen a surge in interest in radiogenomics. Radiogenomics needs the establishment of correlations between quantitative or qualitative imaging aspects and genomic data derived from tissue analysis and other clinical data in order to enable the development of imaging alternatives to genetic testing (81, 82). Radiomics can help to distinguish IDH-mutant co-deleted 1p/19q tumors (oligodendrogliomas) from IDH-mutant non-co-deleted 1p/19q tumors (astrocytomas). Researchers reported that the combination of a near-complete or complete hyperintense signal on a T2 sequence and a hypointense signal on a FLAIR (except a potential hyperintense peripheral rim) possesses a 100% predictive value of IDH-mutant astrocytomas, which was termed as T2-FLAIR mismatch (83). Researchers further verified the specificity of this mismatch for anaplastic astrocytomas and diffuse through a retrospective study containing patients with diffuse oligodendroglioma (IDH-mutant 1p/19q co-deleted), diffuse astrocytoma (IDH-mutant), anaplastic oligodendroglioma (IDH-mutant 1p/19q co-deleted), anaplastic astrocytoma (IDH-mutant), and IDH-WT (Glioblastoma-like) (84). It was revealed that the T2-FLAIR mismatch is present in four of five anaplastic astrocytoma tumors, 34 of 70 diffuse astrocytoma tumors, and 0 of 79 other three types of tumors, confirming the 100% specificity differentiating astrocytomas from other LGGs, which has been further verified in other two studies (85, 86). In addition to the T2-FLAIR mismatch, researchers created a model consisting of T1, T2-weighted FLAIR, and an apparent diffusion coefficient (ADC), and reported that the model can differentiate MGMT methylated tumors from non-methylated tumors with an AUC of 0.925 and 0,902 in the training and validation cohort, respectively. This indicated the efficiency of MRI in the prediction of molecular markers. Employing AI-based MRI can help clinicians to clear changes in molecular markers easily (87, 88). In general, the majority of research employed MRI to predict glioma gene mutations with DL (particularly CNN), RF, least absolute shrinkage and selection operator (LASSO), and SVM technologies to obtain strong predictive performance with an accuracy of greater than 80 - 90%. For example, Choi et al. (57) predicted the IDH genotype with an accuracy of 92.8% and 91.7% in the validation and test sets, respectively, using an RNN application based on dynamic susceptibility contrast MRP from 463 patients with gliomas. The H3- -K27M mutation status prediction model based on CNN features and the SVM classifier was tested by Liu et al. in a group of 55 patients with preoperative T1-magnetization prepared rapid gradient echo (MPRAGE) images MRI, and the results indicated an accuracy of 95% upon fivefold cross-validation (60). For the prediction of deletion of Chromosomal Arms 1p/19q, Akkus and co-workers (62) used a multi-scale CNN based on T1c and T2 pictures from 159 LGGs. Using TL and previously trained 3D-dense-UNets on T2 images, Yogananda and colleagues (58) were able to accurately predict the MGMT promoter methylation status in 247 individuals. Similarly, several studies used CNN and/or RF models to predict molecular markers (such as TERT (61), 7/10 aneuploidies, CDKN2 family mutations (66), receptor tyrosine kinase II (RTKII) (67), and tumor proliferation marker (Ki-67) (63) in glioma patients’ MRI and reached a high degree of accuracy. Additionally, LASSO regression and/or SVM models based on MRI correctly predicted additional molecular indicators such as ATRX mutation (59), TP53 status (64), and vascular endothelial growth factor (VEGF) expression (65).

#### 3.1.3 Response Assessment and Prognosis Prediction

AI has been used in MR imaging sequences to assess response and predict survival in gliomas, excluding the prediction of molecular markers. A significant challenge following chemoradiotherapy is the presence of radiation-induced side effects such as pseudoprogression (PsP), a late benign therapeutic effect that mimics true tumor progression (TTP) at the tumor site or resection margin, which occurs in approximately one-third of GBMs and is usually stable without further treatment (89). Clinicians face significant hurdles because of this discrepancy between PsP and TTP.

SVM has been successfully used to measure response and predict survival in gliomas. Li and co-workers (51) demonstrated a 92% accuracy in differentiating between PsP and TTP after tenfold cross-validation using an SVM classifier based on deep convolutional generative adversarial networks and AlexNet radiomics feature learning from DTI. Conventional MRI data from two institutions, comprising 105 GBMs, was utilized by Ismail and colleagues (52) to distinguish between PsP and TTP. An SVM classifier was utilized to evaluate the test cohort after extracting 30 shape features, and the training and test cohorts had accuracy rates of 91.5% and 90.2%, respectively.

Moreover, some studies have reported the accuracy of AI in predicting glioma prognosis. The cancer imaging archive (TCIA) and local test cohorts were used by Pan et al. to predict the OS using ML techniques with C-indexes of 0.70 and 0.76, respectively, for multiparameter MRI of 152 GBMs (53). When radiomic characteristics were paired with preoperative clinical risk factors (C-index = 0.76 in the TCIA and test cohort), the impact of OS prediction was substantially enhanced. Sanghani and colleagues (56) found that an SVM classifier based on textural characteristics, tumor shape, and volumetric data from conventional MRI was able to accurately predict OS in two- and three-class survival groups following a 5-fold cross-validation. Similarly, Chang and colleagues (55) predicted OS with good accuracy using an RF MRI feature selector and a kernel SVM or neural network classifier. Furthermore, another study demonstrated significant accuracy in identifying survival-relevant high-risk subregions in MRIs from GBMs using the K-means clustering methodology (54).

In summary, ML algorithms are more than 80% accurate in predicting glioma outcomes *via* imaging. One way to improve the efficiency of AI-based MRI in Response assessment and prognosis prediction is to overcome the limitations of MRI. The major disadvantage is that the treatment-related changes can affect MRI results, regardless of the time of evaluation. In this situation, some entities such as radiation necrosis (RN), pseudoprogression (PSP), and pseudoresponse can be introduced (90). Notably, oedema and necrosis caused by postoperative reaction and radio- or chemotherapy could be misinterpreted as disease progression due to the increase in T2/FLAIR signal (91). Therefore, it is suggested to introduce a reliable imaging technique to increase the accuracy of MRI.

#### 3.1.4 Tumor Cell Analysis

Non-enhanced aggressive tumors are difficult to detect with MR enhancement but can be aided by assessing a variety of biophysical characteristics. Hu and colleagues (68) trained a TL model using dynamic susceptibility contrast MR imaging and DTI data from 18 GBMs from a single clinical institution and on 82 image-recorded biopsy samples. With a Pearson correlation value of 0.88 and a mean absolute error of 5.66 percent, the tumor cell density could be predicted. In another study, images from High-Resolution Magic Angle Spinning Nuclear MRS of glioma and control samples were analyzed using an RF model with AUCs of 85.6% and 87.1% to differentiate tumor cells and benign samples from controls and malignant samples (69). Similarly, Fathi Kazerooni and colleagues (70) differentiated subregions of brain gliomas in Fifty-one tissue specimens from 10 patients using conventional MRI, DWI, DTI, intravoxel incoherent motion (IVIM), and dynamic susceptibility contrast MRI. An SVM classifier was used to generate models, and a model based on 15 MRI-based parameters had an AUC of greater than 0.90 for identifying the three subregions (active tumor, infiltrative edema, and normal tissue).

Tumor cell analysis enables the direction of postoperative targeted therapy and the assessment of tumor margins intraoperatively. At the moment, artificial intelligence is still in its infancy. Due to financing and data issues, there are still very few relevant studies available now. Future studies can be conducted to improve the use of AI and the verification of cell analysis.

In general, AI has been extensively applied in glioma MRI, including tumor segmentation and classification, molecular marker prediction, and tumor cell analysis. With the rapid advancement of AI, deep learning in image analysis demonstrates both its advantages and limits. AI will eventually assist in the integration of data from disparate sources (clinical examination, other medical imaging, and pathology) to guide therapy and prognosis.

### 3.2 Positron Emission Tomography

As described above, the application of AI-based MRI shows excellent outcomes in glioma. However, MRI may not always be able to answer three essential questions: evaluation of the initial characterization of the brain lesion, monitoring of therapies to clear changes induced by recurrence/progression and treatment, and evaluation of treatment efficacy (92). Furthermore, one of the main advantages of PET is that the radiotracers used for PET are in most cases independent of disruption of the blood-brain barrier (BBB) as opposed to MRI, which is especially useful in LDH (92, 93). Overall, PET provides insights into glioma that exceed MRI and that can be applied for noninvasive grading, differential diagnosis, mapping the extent of tumor involvement, designing surgery and radiotherapy methods, and prognostic prediction.

PET mainly uses [18F]-fluorodeoxyglucose (18F-FDG) and radioactively labeled amino acids as radioactive tracers. Compared with 18F-FDG, the radioactive labeled amino acid, such as [11C]-methyl-L-methionine (11C-MET), [18F]-fluoro-ethyl-tyrosine (18F-FET), 3,4-dihydroxy-6-[18F]-fluoro-L-phenylalanine (18F-FDOPA) show higher contrast in tumor tissues and normal brain tissues (94). Further, the amino acid PET (AA-PET) can provide additional information on the metabolic characteristics of glioma. These two advantages make the United Cooperative produce guidelines encouraging the use of AA-PET for tumor diagnosis and treatment (95, 96), and the response assessment in neuro-oncology (RANO) group made evidence-based recommendations for the use of PET imaging in the planning and monitoring of radiation therapy for glioma patients (97–99). While the tumor-brain ratio (TBR) is currently the gold standard for estimating neoplastic uptake relative to healthy brain tissue in the majority of centers, tracer uptake dynamics, such as slope and time-to-peak, have been shown to increase diagnostic accuracy (100). Dynamic factors were found to be linked with tumor grade, tumor progression, molecular indicators such as IDH gene alterations, and separating patients with actual and false tumor progression in patients with gliomas (3, 101, 102). The following is a summary of recent AI-based PET studies on glioma diagnosis, treatment, and prognosis (**Table 1**).

**Table 1:** Summary of major studies on AI-assisted PET in Glioma.

Purpose	Ref.	Design ofstudy	Database	Sample size	Performingalgorithm	Modality	Feature	Outcomes (%)	
								Accuracy	Sensitivity/Specificity
Detection and segmentation	Blanc-Durand et al, 2018 (103)	Retrospective	Internal	37 glioma patients	18F-FET PET	CNN Feature	3D U-net CNN	Detection: 100; Segmentation: DSC: 82.31	Detection: 100/100; Segmentation: 88/99
Classification	Kebir et al, 2021 (104)	Retrospective	Internal	7 multiple sclerosis and 34 glioma patients	18F-FET PET	TBR	SVM	91; AUC:94	89/100
Classification	Kong et al, 2019 (105)	Retrospective	Internal	24 lymphoma patients and 53 GBMs	18F-FDG PET	SUV map	Decision tree	90.9-97.4; AUC:97.1-99.8	90.6-98.1/87.5-100
Classification (3-group molecular subtypes)	Matsui et al, 2020 (106)	Retrospective	Internal	217 LGGs 49/58/100 (IDH-wildtype diffuse astrocytoma/IDH-mutant difuse astrocytoma/oligodendroglioma)	MRI, PET, and CT	Image and clinical features	residual network	96.6/68.7 (training/testing)	NA
Discrimination between PsP and TTP	Lohmann et al, 2020 (107)	Retrospective	Internal	34 glioma patients	18F-FET-PET	First-order statistics, shape, and texture, Laplacian-of-Gaussian filtered, wavelet-transformed features	RF	Training/testing: 86/70; AUC:74/74	82/90 (training); 100/40 (testing)
Discrimination between PsP and TTP	Kebir et al, 2020 (108)	Retrospective	Internal	44 glioma patients	18F-FET-PET	TBR and time-to-peak	Linear discriminant analysis	AUC:93	100/80
Discrimination between PsP and TTP	Imani et al, 2014 (109)	Retrospective	Internal	12 grade 2 and 3 gliomas	18F-FDG PET and MRS	Maximal SUV and multiple 2D maps of choline, creatine	SVM	92	80/100
Discrimination between PsP and TTP	Kebir et al, 2017 (110)	Retrospective	Internal	14 HGGs	18F-FET-PET	Textural and conventional features	Clustering based classifier	Positive predictive value: 90	90/75
OS prediction	Papp et al, 2018 (111)	Retrospective	Internal	70 patients with a treatment naive glioma	11C-MET PET	General and higher-order textural features, *in vivo*, ex vivo, and clinical patient information	K-nearest neighbor classifier	90; AUC: 91	88/95
IDH mutation prediction	Li et all, 2019 (112)	Retrospective	Internal	127 consecutive gliomas	18F-FDG PET	Clinical characteristics and the radiomic signature	SVM and multivariate LR	Training/testing: 79.8/83.7; AUC: 91.1/90	78.9/80.4 (training); 92.3/80 (validation)
IDH status prediction	Tatekawa et al, 2021 (113)	Retrospective	Internal	62 treatment-naive glioma patients	Multiparametric MRI and 18F-FDOPA PET	Voxel-wise feature	Two-level clustering and SVM	76; AUC:81	NA
Classification (HGG and LGG) and IDH status prediction	Kebir et al, 2019 (114)	Retrospective	Internal	39 gliomas	11C-MET PET/MRI	TBR	SVM classifier with a linear kernel	Classification: AUC:62; Prediction: AUC:79	NA
MGMT status prediction	Qian et al, 2020 (115)	Prospective	Internal	86 GBMs	18F-FDOPA PET	Shape, tumor intensity and tumor texture features	RF	80	100/33
Ki-67 prediction	Kong et al, 2019 (116)	Retrospective	Internal	123 glioma patients 82/41 (training/testing)	18F-FDG PET	Shape and size, first-order, texture, wavelet, and alternative filtered features	SVM	Training/validation: 81.7/73.2; AUC:88/76	95.6/64.9 (training); 92/43.8 (validation)

AI, artificial intelligence; PET, positron emission tomography; Internal, subjects were recruited from insitutional and/or public through media channels; 18F-FET, [18F]-fluoro-ethyl-tyrosine; CNN, convolutional neural network; DSC, dice similarity coefficient; TBR, tumor-brain ratio; SVM, support vector machine; AUC, area under the receiver operating characteristic curve; 18F-FDG, [18F]-fluorodeoxyglucose; SUV, standardized uptake value; IDH, isocitrate dehydrogenase; MRI, magnetic resonance imaging; CT, computed tomography; NA, not available; PsP, pseudoprogression; TTP, true tumor progression; RF, random forest; 2D, two-dimensional; HGG, high-grade glioma; OS, overall survival; 11C-MET, [11C]-methyl-L-methionine; LR, logistic regression; 18F-FDOPA, [18F]-fluoro-L-phenylalanine; LGG, low-grade glioma; MGMT, methylation of O6-Methylguanine-DNA methyltransferase; GBM, glioblastoma.

#### 3.2.1 Applications for Diagnosis

Glioma misdiagnosis as another lesion can have a significant impact on patient survival, and although MRI is frequently utilized for the first screening, radiological separation of glioma, primary central nervous system lymphoma (PCNSL), and multiple sclerosis remain challenging. PET is an alternative form of imaging that has been used to assess central nervous system disorders (117). As a result, an increasing number of studies have used AI-based PET to aid in the detection and diagnosis of glioma. For example, 18F-FET-PET imaging may differentiate between multiple sclerosis and WHO grade II-IV glioma with a 91% accuracy by using an SVM classifier, according to a study by Kebir et al. (104) In an attempt to identify PCNSL from GBM, Kong et al. (105) used 107 radiomic characteristics from 18F-FDG PET in 77 individuals (24 with lymphoma and 53 with GBM). The decision tree approach algorithm demonstrated great diagnostic performance, according to this study’s findings (accuracy 90.9%-97.4%, AUC 97.1%-99.8%). LGG may be classified into three molecular subtypes based on the WHO’s 2016 categorization of central nervous system malignancies. The mainstay of care for patients with LGG is surgical excision of the tumor followed by postoperative chemoradiotherapy. Their effectiveness, however, is dependent on the tumor’s molecular subtype. Matsui et al. (106) utilized residual networks to predict LGG molecular subtypes using multimodal data from a glioma database, including MRI, PET, and CT, and achieved an overall accuracy of 68.7% for the test dataset.

The above evidence has exhibited the strength of PET in the diagnosis of glioma. However, about 5% of HGG do not show amino-acid tracer uptake (118, 119) and some non-neoplastic lesions, such as vascular malformations, hematomas, inflammatory lesions, and ischemic lesions, can also exhibit unspecific amino-acid uptake (120, 121). Besides, although static 18F-FDG PET has been used for the differentiation of LGG and HGG, overlap can be seen, which may interfere with the judgement (122). Also, static 18F-FDG PET has only a specificity of 56-85% and a sensitivity of 71-80% for the differentiation between LGG and HGG, suggesting the employment of dynamic 18F-FDG PET which can improve the accuracy (95, 123). Therefore, although the application of PET enhances the interpretation of lesions determined by MRI, histological diagnosis and the molecular signature cannot be neglected.

#### 3.2.2 Applications for Treatment

Segmentation is a frequently performed operation in medical imaging; automated segmentation significantly reduces the time required for human segmentation. Segmentation objectives such as radiotherapy plans that define the total or biological tumor volume, and surgical plans that quantify the three-dimensional volume of enhancing tumor and surrounding edema are necessary for accurate assessment and monitoring of tumor response and have also demonstrated some independent prognostic value. A 3D U-Net CNN was employed in 37 glioma patients to detect and segment gliomas using 18F-FET PET with 100% detection accuracy and 82.31% DSC (segmentation) (103).

Although PsP is most frequently noticed within the first 12 weeks following the cessation of radiation and chemotherapy (124), it can develop later (125). Detecting PsP in GBMs continues to be an important clinical problem in radiology since it is necessary to avoid continuing ineffective therapy and discontinuation of beneficial treatment. Kebir et al. (108) developed a model for identifying PsP using 18F-FET PET scans from 44 glioma patients and a linear discriminant analysis model with an AUC of 0.93 was utilized. Lohmann et al. (107) used a model for discriminating PsP from TTP by analyzing 18F-FET PET scans from 34 glioma patients. The patient group was separated into a training and a test cohort. The final model used an RF classifier and attained accuracies of 86% and 70% in the training and test data, respectively. In another study, an SVM classifier was developed on twelve post-therapy patients who underwent 18F-FDG PET and MRS to identify brain glioma progression. The classifier’s sensitivity and specificity for detecting glioma progression were 80% and 100%, respectively, with an accuracy of 0.92 (109).

#### 3.2.3 Applications for Prognosis

PET imaging using radiolabeled amino acid tracers such as 11C-MET and 18F-DOPA is regarded as a potential diagnostic tool for tumor characterization and longitudinal therapy monitoring due to its excellent sensitivity and specificity. Papp et al. (111) assessed the possibility for survival prediction using 11C-MET PET radiomics and clinical patient information in 70 patients with a treatment-naive glioblastoma. The final model incorporated *in vivo*, ex vivo, and clinical patient data and had an AUC of 0.90. Similarly, another study (114) showed a good AUC for IDH status prediction using an SVM classifier while assessing 11C-MET PET scans from glioma patients. Based on 18F-DOPA PET images, RF and SVM models correctly predicted MGMT status (115) and tumor proliferation marker (Ki-67) (116). Additionally, several studies employ a combination of multimodal imaging and machine learning methods to predict tumor genetic markers. For example, Tatekawa et al. (113) performed a radiomics analysis based on multiparametric MRI and 18F-DOPA PET images for the prediction of the IDH status in 62 treatment-naïve glioma patients, a SVM model achieved an AUC of 81% after leave-one-out cross-validation (LOOCV).

Taken together, feature-based PET radiomics has shown promise in the field of neuro-oncology, allowing for the evaluation of more data at a reasonable cost. However, the majority of existing research is retrospective in nature, with insufficient sample sizes and no available database. ML is a technique for fitting statistical models, and its outcomes are sample size-dependent.

### 3.3 Spectral Imaging

Glioma is defined by its proclivity for metastasis and heterogeneity. Due to the lack of specificity of early clinical signs, the majority of glioma patients are frequently overlooked, resulting in patients missing the best treatment window. Histopathology has evolved into the gold standard for classification and diagnosis, whereas molecular pathology has gained increasing attention in the diagnosis and classification of glioma. With the advancement of molecular biology and molecular pathology in 2016, the WHO categorization of recombinant central nervous system malignancies is beneficial for early detection and accurate therapy (3). Spectral imaging is a potential tool for assisting in the histopathological study of cancer samples that contain molecular information. Imaging can be employed for real-time intraoperative evaluation, allowing for earlier detection and more precise intraoperative resection, which is critical for patient survival (126).

#### 3.3.1 Infrared spectroscopy

Infrared spectroscopy (IS) is a non-invasive and quick measuring technique used to characterize biological samples and their constituents qualitatively and quantitatively by quantitative detection of molecule internal vibration patterns (127, 128). Several studies have coupled human serum IS with ML methods to identify glioma (129–131). Hands et al. (131) extracted 130 features from Fourier-transform IS pictures of blood samples from 433 individuals with or without glioma. The final SVM classifier model has a sensitivity and specificity of 91.5% and 83.0%, respectively, for detecting glioma. In this test, SVM and RF outperformed other classifiers. Another model was constructed using partial least squares discriminant analysis and synthetic minority over-sampling to classify GBM multiforme and lymphoma from 765 serum samples. The result has a sensitivity of 90.1% and a specificity of 86.3%, respectively (132).

Furthermore, the combination of IS with a microscope enables the spatial distribution of proteins, lipids, nucleic acids, and other compounds in tissue samples to be examined. Peng et al. (133) used Fourier transform infrared microscopy to study 9360 spectra from the tissue of 77 glioma patients. This study employed artificial neural networks to categorize gliomas (HGG and LGG) with higher than 98% accuracy, specificity, and sensitivity. For estimating the secondary structure of proteins, Surowka et al. (134) employed infrared micro-spectroscopy spectral range. ANNs were employed to generate the models, and the accuracy was improved to less than 5%.

#### 3.3.2 Raman Spectroscopy

RS is a label-free method that generates spectra by detecting and measuring Raman scattering using narrow-band laser excitation and sensitive spectrometers. For stereotactic brain tumor biopsy, *in vivo* tumor infiltration detection, intra-operative histopathology diagnosis, and molecular categorization, it gives quantitative biochemical information regarding the molecular composition (135–138).

For the creation of a model to grade glioma, Zhou et al. (139) employed label-free visible resonance RS spectra from 125 histologically normal human brain tissues and glioma tissues. The SVM model was able to discriminate normal, LGGs, and HGGs 75.1% of the time. Besides, Pekmezci and coworkers (140) used RS spectral data to differentiate the phenotypes of T-cells and monocytes following incubation with a medium conditioned by GBM stem cells with a variety of genetic backgrounds in three human GBM cell lines. The linear discriminant analysis model was generated using 67% of the dataset (training set) and then verified against 33% of the dataset (test set). The SVM produced sensitivities and specificities of greater than 70% and 67% in the validation and independent test sets, respectively.

#### 3.3.3 Fluorescence Spectroscopy

FS offers a comprehensive array of detection tools and procedures for high-grade gliomas that accumulate the endogenous biomarker protoporphyrin IX following exogenous treatment of 5-aminolevulproic acid, boosting tumor tissue fluorescence and directing surgical intervention (141, 142). In ten glioma patients, Valdés and colleagues (143) assessed the possibility of combined FS and reflectance spectroscopy *in vivo* optical data for diagnostic performance during surgery. The SVM model attained an accuracy of 94%. Leclerc et al. (144) used spectral characteristics analysis based on FS to identify healthy tissue from margin tissue in 50 samples from ten patients. A completely automated clustering technique obtained a diagnostic accuracy of 77% in predicting healthy tissues from margin tissues.

#### 3.3.4 Hyperspectral Imaging

HI measures the diffuse reflectance of tissue surfaces to generate spectral characteristics that contain both spatial and spectral information (145). Recently, HI has been utilized to identify and diagnose illnesses characterized by alterations in cellular biochemical pathways (146). Urbanos et al. (147) classified tumor tissue in a set of 12 HGGs using thirteen *in-vivo* hyperspectral photos (healthy tissue, tumor, venous blood vessel, arterial blood vessel, and dura mater). Overall accuracies for the three models (RF, SVM, and CNN) ranged from 60% to 95% depending on the training settings. Similarly, Manni and coworkers (148) classified tumor tissue (tumor, healthy tissue, and blood vessels) in 16 tumor patients using 26 *in-vivo* hyperspectral pictures. The hybrid 3D-2D CNN models achieved an overall accuracy of 80%. Ortega et al. (149) employed 527 high-resolution pictures to detect GBM in non-tumor brains and GBM samples from 13 individuals. The CNN models had an average sensitivity and specificity of 88% and 77%, respectively.

In conclusion, these investigations demonstrate that spectral image-based AI is beneficial for diagnosing and applying glioma tissue samples intraoperatively. Due to spectrum imaging’s unpopularity, there are few studies and their sample sizes are modest. As a potential intraoperative quick diagnostic method, more research may be directed toward developing applicable AI software.

#### 3.3.5 Magnetic Resonance Spectroscopy Imaging

MRSI is a non-invasive technique for evaluating the spatial distribution of metabolic changes in the brain (150, 151). It can provide information on neuron integrity, neurotransmitter levels, and specific biological information like as cell membrane turnover, cell density, and cell proliferation, complementing the structural pictures of conventional MRI. The measured brain MRSI includes complicated signals corresponding to several overlapping peaks of various metabolites, baselines from various macromolecules and lipids, as well as noise and distortion (152). By measuring the concentration levels of specific metabolites, *in vivo* and *in vitro* MRSI studies (153, 154) of the brain can indicate tumor kind, grade, or invasion and distinguish tumor growth from post-radiation necrosis.

In one work, the SVM classifier and the minimum redundancy maximum relevance algorithm were used to predict glioma grade based on twenty-six metabolic characteristics from the preoperative MRSI. This model attained AUCs of 0.825% in the training set and 0.820% in the validation set (50). In another study, the dictionary pair learning approach was designed to evaluate glioma based on 150 spectra; its overall accuracy was 0.9778 (155). For glioma treatment response, in a research of 29 control mice and 34 TMZ-treated mice, the performance of an SVM classifier with a linear kernel over the number of sources picked for the MRS image data was able to identify between treated and untreated mice with GBM with an accuracy of over 80% (156).

## 4 Limitations and Future Considerations

Simultaneous advancements in image processing technology (MRI, PET, and spectral imaging) and AI, particularly in machine learning and deep learning, have enabled these data-rich patterns to provide diagnostic and guidance information for glioma patients in a non-destructive manner. The majority of these technologies have demonstrated a moderate to a high degree of accuracy. However, some constraints must be solved before these novel predictive analytics algorithms can become widely used in glioma diagnosis and therapy.

Initially, the use of AI in glioma is still in its infancy, with the majority of research being retrospective with limited sample size. It is difficult to validate the safety and reliability of these models in clinical practice. The present medical scientific environment requires data sharing, data management, data standards, and interoperability. Additionally, as machine learning continues to change the area of healthcare, it has posed a variety of challenging ethical problems. If misdiagnosis happens in the use of AI, issues of moral and legal accountability must be addressed (157). Another difficulty is the “black box” aspect of AI technology, which leaves developers and consumers in the dark about how a computer generates its results and lacks interpretability and transparency (158). Radiomics is an AI-assisted technique that will confront the same challenge in the diagnosis and treatment of glioma, which may restrict clinical application (159). However, there are several directions to modify this problem. Applications of radiomics in glioma belong to more deterministic domains. The AI-calculated result assigns the images a relevant annotation (such as a certain gene mutation, the prognosis of the disease is good or bad). Using other patient samples, medical specialists can objectively validate the tags assigned to the images (17, 160), which could reduce the impact of the “black box”. Besides, a new generation of AI which has better reliability, interpretability, accountability, and transparency than black-box AI is worth investing in to overcome the “black box” dilemma. For example, Jia et al. created visualizing surrogate decision trees of convolutional neural networks with python (161).

**Figure 3:**
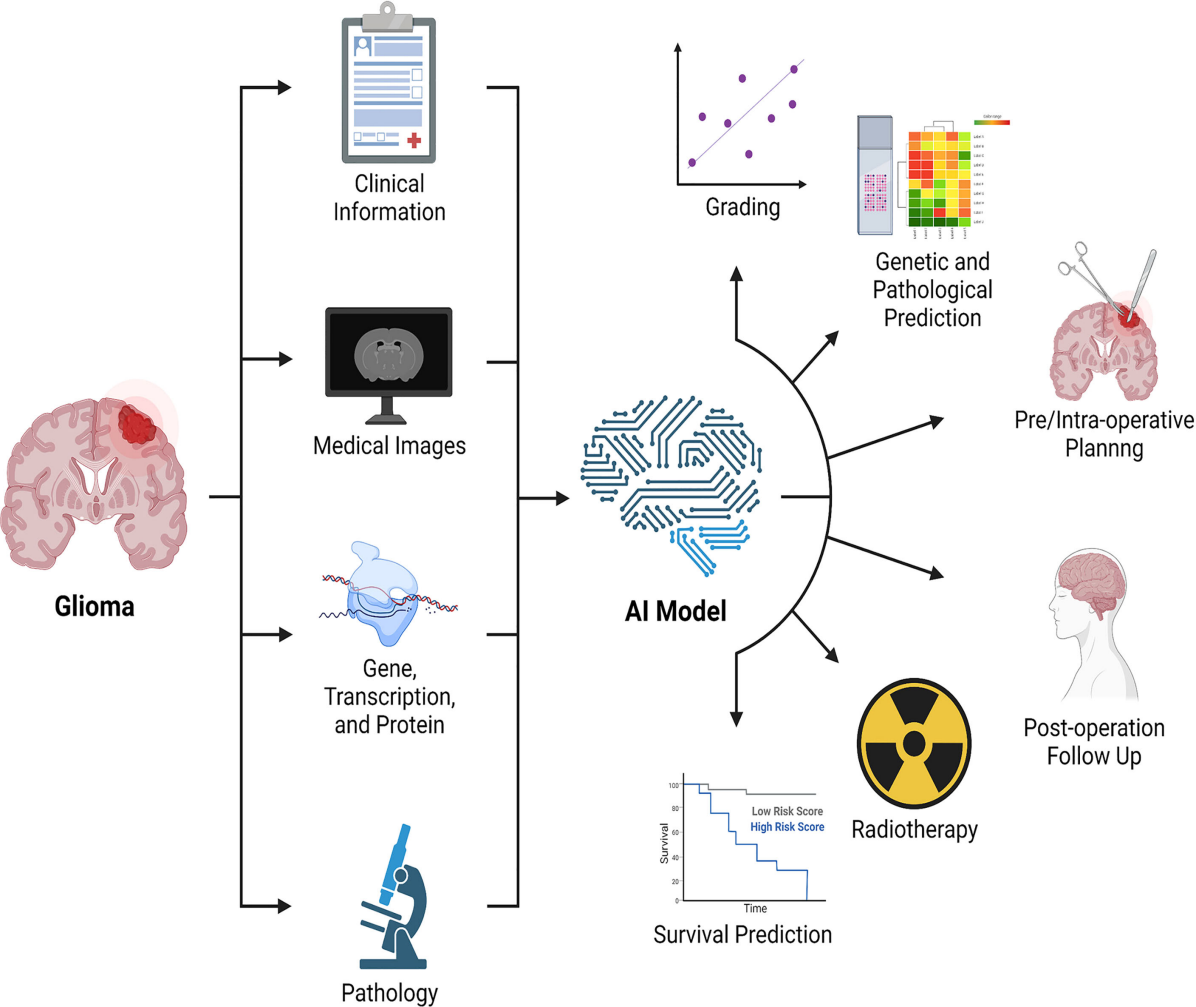
Combination of multi-omics analysis and artificial intelligence. Artificial intelligence integrates clinical data, medical imaging, genomics, transcriptomics, proteomics, and pathology, among other things, to enable the application of multiple omics in glioma, with the potential to detect and evaluate lesions, promote treatment, and predict treatment response and prognosis. AI: artificial intelligence.

Notable also is the fact that the research described above consists of analyses of a single type of data, a technique known as single-omics analysis (radiomics). However, single-omics data analysis has limits, and it is not apparent which data types should be used to reflect clinical characteristics. A qualified physician should evaluate not only the type of tumor when diagnosing and treating patients, but also the pathology, genes, medical imaging of the tumor, and clinical aspects of the patients. Along with radiomics, genomics, transcriptomics, and high-throughput proteomics are all examples of “omics” techniques that provide data for the examination of molecular constituents. In a general sense, the multi-omics analysis consists of three components: input data, technique, and output data (162). Multiple omics analysis is crucial in neuro-oncology research with limited sample sizes. While the multi-omics analysis may analyze several types of data in parallel for humans, human-dependent multi-omics analysis is not repeatable or interpretable. This challenge can be tackled by integrating machine learning techniques (163) such as multi-modal learning, multi-task learning, representational learning, semi-supervised learning, and automated feature acquisition. Recently, radiomics and radiogenomics (81, 82, 164) have received considerable interest, as have various studies that analyze both radiographic and histological pictures (165). The multi-omics analysis enables us to acquire a more complete knowledge of the illness to improve clinical applications such as determining therapy efficacy, predicting prognosis, and identifying the optimal treatment (**Figure 3**).

In general, AI will show its superiority and larger-scale research will be carried out. Clinicians need to increase interaction with engineers to complement knowledge gaps in both fields. In the future, multidisciplinary collaboration remains a crucial aspect. Researchers will be able to combine multi-omics data to discover drugs and assess treatment effects, predict prognosis, and discover the best treatment for each patient. Finally, while AI has played a huge role in the medical field, AI still can’t replace doctors.

## 5 Conclusion

This review retrospectively summarizes some sample studies on the applications of AI in the diagnosis and treatment of glioma using MRI, PET, and spectral imaging. AI is advancing at a breakneck pace and is emerging as a viable tool for medical picture analysis. However, we should be mindful that the implementation of AI in clinical practice is not without flaws. While we are continually working to improve the accuracy of AI, we should not rely excessively on it, as it cannot replace the clinician.

## Author Contributions

JX, YM, KQ, MC, ZD and GN wrote this paper. WT, SL, CW, TC, FQ, MC, ZD and GN reviewed this paper. All authors read and approved the final manuscript.

## Funding

This work was supported by Natural Science Foundation of Zhejiang Province (NO. LQ22H160003 and NO. Y22H185692).

## Conflict of Interest

The authors declare that the research was conducted in the absence of any commercial or financial relationships that could be construed as a potential conflict of interest.

## Publisher’s Note

All claims expressed in this article are solely those of the authors and do not necessarily represent those of their affiliated organizations, or those of the publisher, the editors and the reviewers. Any product that may be evaluated in this article, or claim that may be made by its manufacturer, is not guaranteed or endorsed by the publisher.

## Abbreviations

WHO, World Health Organization; LGG, low-grade glioma; HGG, high-grade glioma; OS, overall survival; GBM, glioblastoma; AI, artificial intelligence; MRI, magnetic resonance imaging; PET, positron emission tomography; ML, machine learning; SVM, support vector machine; LR, logistic regression; RF, random forest; DL, deep learning; ANN, artificial neural network; CNN, convolutional neural network; DNN, deep neural network; RNN, recurrent neural network; DA, deep auto-encoder; DBN, deep belief network; DBM, deep Boltzmann machine; GAN, generative adversarial network; VAE, variation auto-encoder; VGG, Visual Geometry Group; TL, transfer learning; CT, computed tomography; ROI, region-of-interest; AUC, area under the receiver operating characteristic curve; DSC, dice similarity coefficient; T1, T1-weighted; FLAIR, fluid-attenuated inversion recovery; T2, T2-weighted; DWI, diffusion weighted imaging; DTI, diffusion tensor imaging; DKI, diffusional kurtosis imaging; PWI, perfusion weighted imaging; ASL, arterial spin labeling; DCE, dynamic contrast-enhanced; MRS, magnetic resonance spectroscopy; BraTS, Brain Tumor Segmentation; T1c, T1-weighted contrast-enhanced; XGBoost, eXtreme Gradient Boosting; IDH, isocitrate dehydrogenase; MGMT, methylation of O6-Methylguanine-DNA methyltransferase; EGFR, epidermal growth factor receptor; TERT, telomerase reverse transcriptase promoter; CDKN, cyclin-dependent kinase inhibitor; ATRX, alpha thalassemia/mental retardation syndrome X-linked; TP53, tumor protein 53; LASSO, least absolute shrinkage and selection operator; MPRAGE, Magnetization Prepared Rapid Gradient Echo; RTKII, receptor tyrosine kinase II; VEGF, vascular endothelial growth factor; PsP, pseudoprogression; TTP, true tumor progression; TCIA, imaging archive; IVIM, intravoxel incoherent motion; 18F-FDG, [18F]-fluorodeoxyglucose; 11C-MET, [11C]-methyl-L-methionine; 18F-FET, [18F]-fluoro-ethyl-tyrosine; 18F-FDOPA, [18F]-fluoro-L-phenylalanine; AA-PET, amino acid PET; RANO, response assessment in neuro-oncology; TBR, tumor-brain ratio; TTP, time-to-peak; PCNSL, primary central nervous system lymphoma; LOOCV, leave-one-out cross-validation; IS, infrared spectroscopy; RS, Raman spectroscopy; FS, fluorescence spectroscopy; HI, hyperspectral imaging; MRSI, agnetic resonance spectroscopy imaging.
