# Supplementary material for: Applications of Artificial Intelligence Based on Medical Imaging in Glioma: Current State and Future Challenges
Source: Front Oncol. 2022 Jul 27;12:892056. doi: 10.3389/fonc.2022.892056 (PMC9363668; doi:10.3389/fonc.2022.892056)
Supplement: Supplementary file 1 [file Table_1.docx]

**Table 1 Summary of major studies on AI-assisted MRI in Glioma**

| Purpose | Ref. | Design of  study | Database | Sample size | Performing  algorithm | Modality | Feature | Outcomes (%) | |
| --- | --- | --- | --- | --- | --- | --- | --- | --- | --- |
|  |  |  |  |  |  |  |  | Accuracy | Sensitivity/Specificity |
| Tumor segmentation and classification | | | | | | | | | |
| Segmentation | Liu et al, 2021[1] | Retrospective | BraTS2017, BraTS2018 and BraTS2019 | 329/351/460 glioma cases (BraTS2017/BraTS2018/BraTS2019) | Context-Aware Network | T1, T1c, T2 and FLAIR | High dimensional and discriminative features with contexts | Enhancing tumor/whole tumor/tumor core  BraTS2017 Dice: 72.8/89.2/82.1;  BraTS2018 Dice: 76.7/89.8/83.4;  BraTS2019 Dice: 75.9/88.5/85.1 | Enhancing tumor/whole tumor /tumor core  BraTS2017:  Sensitivity: 80.7/92.4/87.0  Specificity: 99.7/99.3/99.6  BraTS2018: NA  BraTS2019: NA |
| Segmentation | Fu et al, 2021[2] | Retrospective | BraTS 2019 | 259 GBM patients | 3D DenseNet | T1, T1c, T2 and FLAIR | DL feature | Single-path DenseNet:  DSC: 91.1;  Multi-path DenseNet:  DSC: 92.2 | NA |
| Segmentation | Khosravanian et al, 2021[3] | Retrospective | BraTS 2017 | 285 glioma patients | Superpixel fuzzy clustering and lattice Boltzmann method | FLAIR | Superpixel and histogram | Dice: 93 | 91.83/99.72 |
| Segmentation | Park et al, 2021[4] | Retrospective | Internal | Training dataset: 238 pre-treatment GBMs; Internal validation dataset: 40 pre- and 53 post-treatment GBMs; External validation dataset: 58 pre- and 44 post-treatment GBMs | 3D U-Net | T1, T2, FLAIR, T1c, DWI, and dynamic susceptibility contrast imaging | T1c and histogram features from ADC and CBV mapping | AUC: 81/78 (internal and external validation set) | NA |
| Segmentation | Rahmat et al, 2020[5] | Retrospective | Internal | 80 GBMs | CNN | DTI, T1c, T2/FLAIR, and PWI | CNN feature | DSC: (p/q/FLAIR/T1c mask): 71/66/78/82 | NA |
| Segmentation | Amin et al, 2019[6] | Retrospective | BraTS 2013, BraTS 2015, and  internal | 30/273/86 glioma patients (BraTS 2013/BraTS 2015/ local dataset) | SVM, RF, Decision Tree, Naive Bayes, and K-nearest neighborhood | FLAIR and T2 | Fused texture feature | BraTS 2013: 93,  AUC/DSC: 96/96;  BraTS 2015: 97,  AUC/DSC: 98/98;  local dataset: 90,  AUC/DSC: 77/95 | BraTS 2013: 100/92  BraTS 2015: 100/97  local dataset: 91/90 |
| Prediction of surgical resectability | Marcus et al, 2020[7] | Prospective | Internal | 135 GBM patients | ANN | pre- and post-operative T1c MRI | ANN feature | 83;  AUC: 87 | 59/92 |
| Delineation of the clinical target volume | Shusharina et al, 2020[8] | Retrospective | Internal | 206 glioma and GBM cases | U-Net CNN | CT, T1c and T2/ FLAIR. | CNN feature | DSC:91-97 | NA |
| Classification | Basha et al,  2021[9] | Retrospective | Dataset1:  IXI/ REMBRANDT/TCGA-GBM/TCGA-LGG  Dataset2:  internal | Dataset1: 8.000 healthy and 8.000 glioma images  Dataset2: 1.636 glioma, 1.636 meningioma, and 1.636 pituitary images | CNN + enhanced Harris Hawks optimization | Axial T1 images | CNN feature | Dataset1:  healthy/grade II/ III/ IV:97/98/98/98  Dataset2:  glioma/meningioma/pituitary:  98/99/99 | Dataset1:  95/97 (healthy)  94/98 (grade II)  90/98 (grade III)  92/98 (grade IV)  Dataset2:  96/97 (glioma)  97/98 (meningioma)  97/100 (pituitary) |
| Classification  (meningiomas, gliomas, and pituitary tumors) | Abdelaziz et al, 2020[10] | Retrospective | Internal | 233 patients with 3064 MRI images | Residual networks | T1c | CNN feature | 99 | 99/NA |
| Classification  (GBM and PCNSL) | Xia et al, 2021[11] | Retrospective | Internal | 289 patients with PCNSL or GBM | Multi-parametric CNN | 3.0 T axial T1c, FLAIR, and DWI | CNN feature | 89.9;  AUC: 96.4 | 93.4/86.7 |
| Classification  (GBM and PCNSL) | Yun et al, 2019[12] | Retrospective | Internal | 123/30/42 (training/internal/external) image sets | Multilayer perceptron network | T1c and DWI | First-order, texture, and wavelet features | AUC (training/internal/external validation): 99.4/99.1/94.7 | 100/100 (training)  100/100 (internal)  92.9/82.1 (external) |
| Classification (GBM and solitary brain metastasis) | Shin et al, 2021[13] | Retrospective | Internal | 450/48/100/143 (training/validation/internal/external test) | ResNet-50 | Preoperative T1c and T2 | CNN feature | Internal and external test sets:  89/85.9,  AUC:88.9/83.5 | Sensitivity: 93.9/88.9 (internal and external test sets) Specificity: NA |
| Classification  (GBM and brain metastasis) | Qian et al, 2019[14] | Retrospective | TCGA and internal | 227/185 (training/test) patients | SVM and LASSO | T1, T1c, T2 | Shape, first-order intensity statistics, texture, square, square root, logarithm, exponential,  Laplacian of  Gaussian, and wavelet features | 83;  AUC: 90 | 80/87 |
| Classification (GBM and LGG) | Ning et al, 2021[15] | Retrospective | TCIA and internal | 567 patients (211 GBMs and 356 LGGs)  training (n=186), validation (n=47), and testing (n=334) | CNN and SVM | T1 and T2/FLAIR | Global radiomics and local deep features | AUC:94/88 (validation/testing) | 86/92 (validation)  88/81 (testing) |
| Classification (normal, HGG, and LGG) | Al-Saffar et al, 2021[16] | Retrospective | TCIA | 1467 (axial plane) MR images of 160 patients | Multiple eigenvalues selection,  and radial basis function SVM. | MRI images | Intensity based and gray level  co-occurrence matrix-based features | 91.02 | 86.52/94.26 |
| Tumor Detection and Classification (diffuse midline glioma of the pons,  medulloblastoma,  pilocytic astrocytoma, and ependymoma) | Quon et al, 2020[17] | Retrospective | Internal | 617 posterior fossa tumors children and 199 controls | Modified ResNeXt-50-32x4d architecture | T2 images | CNN feature | 92 (classification)  AUC:99 (detection) | NA (classification)  96/100 (detection) |
| Histological diagnosis and molecular subtyping prediction | Luo et al, 2021[18] | Retrospective | Internal | 655 glioma patients,  188/411/56  (training/validation/independent testing) | 3D U-net | T1c and T2/FLAIR | High-throughput network features | Histological diagnosis: 89.8/83.9 (validation/testing);  molecular subtyping prediction: 86.1/80.4 (validation/testing) | NA |
| Transcriptome subtypes Prediction | Le et al, 2021[19] | Retrospective | TCGA-GBM and Ivy  GBM Atlas | 86/34 (training/validation) GBMs | XGBoost | T1, T1c, T2, and T2-FLAIR | Intensity information, image derivative, geodesic information, texture features, and glioma image; segmentation and registration posterior probability maps | 70.9/73.3/88.4/88.4 (classical, mesenchymal, neural, and proneural subtypes) | classical: 25/84.8;  mesenchymal: 70.6/75;  neural: 45.5/94.7;  proneural: 66.7/95.4 |
| Molecular subtyping of gliomas prediction | Lu et al, 2018[20] | Retrospective | TCIA and internal | 214/70 (training/validation) gliomas patients | SVM | T1c, T2/FLAIR, T2, and  DWI | First-order, texture,  local binary pattern, scale invariant feature transform, shape and size features | 81.1 | NA |
| Molecular subtyping of gliomas prediction | Diamandis et al, 2018[21] | Prospective | Internal | 55 glioma patients and 10 non-glioma patients | RF | MRS | First order features of metabolite | 94.2 | NA |
| Grading | Zhuge et  al, 2020[22] | Retrospective | TCIA and  BraTS2018 | 210 HGGs and 105 LGGs | CNN | T1, T1c, T2, and T2/FLAIR | CNN feature | 2D Mask R-CNN: 96.3;  3D ConvNet method: 97.1 | 2D Mask R-CNN: 93.5/97.2; 3D ConvNet method: 94.7/96.8 |
| Grading | Zhang et al, 2020[23] | Retrospective | Internal | 43 LGGs and 65 HGGs | CNN | DTI | Convolutional deep features, texture features, and shape/morphological features  from FA and MD maps | Classifying LGGs from HGGs:  94, AUC:93;  classifying grade III from IV:  98, AUC:99 | Classifying LGGs from HGGs: 98/86;  classifying grade III from IV:  98/100 |
| Grading | Takahashi  et al, 2019[24] | Retrospective | Internal | 44/11 gliomas (training and testing) | LR and SVM | DKI and DTI | Avelet-transformed, texture and  global (histogram-based) features | LR: 91;  AUC: 90;  SVM: 91;  AUC: 93 | NA |
| Grading | Tian et al, 2018[25] | Retrospective | Internal | 153 patients | SVM | 3.0T T1, T1c, T2, DWI and 3D ASL images. | Texture features and histogram parameters | Classifying LGGs from HGGs: 96.8, AUC:98.7;  classifying grades III from IV: 98.1, AUC:99.2 | Classifying LGGs from HGGs:  96.4/97.3;  classifying grades III from IV: 98.7/97.4 |
| Grading | Choi et al, 2020[26] | Retrospective | Internal | 386 astrocytomas | CNN | Dynamic susceptibility contrast -enhanced and dynamic susceptibility contrast MRI | Dynamic susceptibility contrast-derived background parmacokinetic parameters, DL fearture | AUC: 88/87/73 (volume transfer constant/fractional volume of extravascular extracellular space/vascular plasma space) | Volume transfer constant：77.6/90;  fractional volume of extravascular extracellular space: 91.4/70;  vascular plasma space：57.8/90 |
| Grading | Qi et al, 2019[27] | Retrospective | Internal | 112 LGGs and HGGs: 74/38 (training/validation) | SVM | MRS | Metabolic features | AUC: 82.5/82 (training /validation) | 74.2/81.4 (training);  89.5/63.2 (validation) |
| Response assessment and prognosis prediction | | | | | | | | | |
| Discrimination between TTP and PsP | Akbari et al, 2020[28] | Retrospective | Internal | 83 GBMs  40/23/20  (discovery/replication /interinstitutional cohort) | CNN and SVM | T1, T1c, T2, T2/FLAIR, DTI, dynamic susceptibility contrast MRI | Quantitative imaging phenomic features | Leave-one-out cross-validation 84/87 (TTP/PsP);  discovery/replication cohort 78/87 (TTP/ PsP);  interinstitutional cohort: 75 | Leave-one-out cross-validation:  TTP: 80/89.29,  PsP: 80/88.68;  discovery/replication cohort:  TTP: 83.33/72.73,  PsP: 75/89.47 |
| Discrimination between TTP and PsP | Elshafeey et al, 2019[29] | Retrospective | Internal | 98 GBMs | SVM | Dynamic susceptibility contrast and dynamic contrast-enhanced perfusion MRI | Radiomic features from pharmacokinetic Ktrans parameter and rCBV maps | 90.82;  AUC: 89.10 | 91.36/88.24 |
| Discrimination between PsP and TTP | Li et al, 2020[30] | Retrospective | Internal | 84 GBM patients | Deep convolutional GAN, AlexNet, and SVM | DTI | DL feature | 92  AUC:94.7 | NA |
| Discrimination between PsP and TTP | Ismail et all, 2018[31] | Retrospective | Internal | 59/46 (training/testing) GBM patients | SVM | T1 and T2/FLAIR | Shape feature | 91.5//90.2 (training/testing) | NA |
| Radiotherapeutic response prediction | Pan et al, 2020[32] | Retrospective | TCIA and  internal | TCIA:122 GBM patients, 82/40/30 (training/validation/independent test) | RF | T1, T1c, T2, and T2/FLAIR | Shape, intensity, and texture features | TCIA/independent test cohorts: 93.9/90;  AUC: 98/93.7 | TCIA: 94.6/93.2;  independent test cohorts: 82.9/100 |
| Survival-relevant high-risk subregion identification | Zhang et al, 2020[33] | Retrospective | TCGA and TCIA | 104 GBM patients | Multiple  instance learning | T1c, T1, T2, and FLAIR | Texture features | 87.88 | 85.71/89.47 |
| OS prediction | Soltani et al, 2021[34] | Retrospective | BraTS 2019 | 101 gross total  resection status patients, 99 unavailable resection status  patients | ANN and RF | MRI images | First order, shape  and texture features | Gross total resection status: 78；  unavailable resection status:  66 | Gross total resection status: 80/60；  unavailable resection status：66/66; |
| OS prediction | Chang et al, 2019[35] | Retrospective | Internal | 12 recurrent malignant glioma patients | Univariate Cox regression, RF, neural networks, and kernel SVM | The pre-treatment, one-week post-treatment, and two-month post-treatment T1 and T2/FLAIR | Delta-features | One-week delta-features:  AUC: 88.9 | NA |
| OS  prediction | Sanghani et al, 2018[36] | Retrospective | BraTS 2017 | 163 GBM patients | SVM | T1, T1c, T2 and FLAIR | texture features, tumor shape and volumetric features | 2-class and 3-class:98.7 and 88.95 | NA |
| Glioma survival associations | Emblem et al, 2015[37] | Retrospective | Internal | 235 patients | SVM | T1c, T2, FLAIR, and dynamic susceptibility contrast-enhanced  MRI | rCBV histograms | 6-month and 1-, 2-, and 3-year survival:  AUC: 79.4/76.2/80.6/85.1 | 6-month: 78/81;  1-year: 76/76;  2-year: 76/85;  3-year: 85/86 |
| Molecular marker prediction | | | | | | | | | |
| Prediction of IDH genotype | Choi et al, 2019[38] | Retrospective | Internal | 395/50/18 (training/validation/testing) | RNN | T1, T2, T2/FLAIR, T1c and dynamic susceptibility contrast perfusion MRI | DL feature | Validation set:  92.8, AUC: 98;  test set:  91.7, AUC:95 | Validation set: 92.6/93.1;  test set: 92.1/91.5 |
| Prediction of IDH genotype | Tan et al, 2019[39] | Retrospective | Internal | 74/31 (training/validation cohorts) astrocytomas (Grades II–IV) | SVM | T1c, T2/FLAIR, and DWI | Shape and size, first-order statistics, textural features | Training:  81.1, AUC: 90.1;  validation:  80.6, AUC:88.8 | Training: 91.7/71.1  validation: 93.3/81.3 |
| IDH1  mutation  status in glioma prediction | Chang et al, 2018[40] | Retrospective | Internal and TCIA | 496 patients,  training, validation, and  testing sets: 8:1:1 ratio | Residual CNN | FLAIR, T2, T1, and T1c | CNN feature | Training:  82.8, AUC:90;  Validation:  83, AUC:93;  Tset:  85.7, AUC:94 | NA |
| Gliomas grading and IDH-mutation status prediction | Bisdas et al, 2018[41] | Prospective | Internal | 37 patients | SVM | DKI | Texture biomarkers | Grading:  78.1, AUC: 79;  Prediction：  83.8, AUC: 88 | Grading: 77/79;  prediction: 92/64 |
| Predicting IDH genotype in WHO grade II/III gliomas | Eichinger et al, 2017[42] | Retrospective | Internal | 79 patients  59/20 (training/validation) | NNet | DTI | Local binary pattern texture features from B0 and FA maps | Training:  92, AUC: 92.1;  Validation:  95, AUC:95.2 | NA |
| MGMT promoter methylation status prediction | Yogananda et al, 2021[43] | Retrospective | TCGA and TCIA | 247 subjects | 3D-dense-UNets + TL | T2 | 3D patches | 94.73;  AUC: 93 | 96.31/91.66 |
| Co-occurrence of IDH mutation and MGMT methylation prediction | Zhang et al, 2021[44] | Retrospective | Internal | 162 glioma patients | Tree-based pipeline optimization tool | 3D T1c, and FLAIR | Shape and texture features | 89.4;  AUC:95.1 | 81.1/94 |
| Prediction of ATRX mutation | Li et al, 2018[45] | Retrospective | TCGA and CGGA | 63/32/91 glioma patients  (training/validation/external validation) | LASSO regression and SVM | T2 | First order, shape, size, textual and wavelet features | Training, validation and external validation:  95.2/93.8/76.9,  AUC: 94/92.5/72.5 | Training: 92/89.5;  validation: 83.3/100;  external validation: 57.1/85.7 |
| Prediction of IDH1 mutation and ATRX expression loss | Ren et al, 2019[46] | Retrospective | Internal | 57 LGG patients | SVM | 3.0T MRI / 3D ASL, T2/FLAIR, and DWI | First-order  statistics, shape/size-based, textual, and wavelet features | IDH1: 94.74/93.1;  ATRX (-): 91.67/92.6 | IDH1: 100/85.71;  ATRX (-): 94.74/88.24 |
| H3-K27M mutation prediction | Su et al, 2020[47] | Retrospective | Internal | 100 patients with midline glioma, 75% training and 25% testing, independent cohort of 22 patients | Tree-based pipeline optimization tool | FLAIR | First-order, shape, and texture features | Testing:  80, AUC:90.3;  validation:  86.4, AUC:85 | Testing: 63.6/92.9;  validation: 80/91.7 |
| Prediction of genotype (H3- -K27M) mutation status | Liu et al, 2018[48] | Retrospective | Internal | 38/4/13 brainstem gliomas (training/validation/testing) | CNN and SVM | Preoperative T1-MPRAGE images | CNN feature | 96.52;  AUC: 95.3 | 96.67/NA |
| Prediction of TERT promoter mutations | Fukuma et al, 2019[49] | Prospective | Internal | 108 IDH mutation glioma patients | CNN and SVM | T1, T2, T1c, and FLAIR imaging | Texture features | 84 | NA |
| Predicting deletion of chromosomal arms 1p/19q in LGG | Akkus et al, 2017[50] | Retrospective | Internal | 159 LGGs | CNN | Preoperative T1c and T2 images | CNN features | 87.7 | 93.3/82.22 |
| Ki-67 prediction | Gates et al, 2019[51] | Prospective | Internal | 52 biopsies from 23 glioma patients | RF | T2, DTI, and dynamic susceptibility contrast | Quantitative maps from T2, FA, CBF and Ktrans | Mean square error:  3.5, R2:75 | NA |
| Predicting TP53 status in LGG | Li et al, 2018[52] | Retrospective | Internal | Training (n=180) and validation (n=92) | LASSO and SVM | T2 | First-order statistics or related wavelet, shape, size-based, and textural features or related wavelet features | Training set:  80, AUC:89.6;  validation set:  70.7, AUC:76.3 | Training set: 80.3/84.6  validation set: 62.2/85.1 |
| Predicting VEGF expression | Sun et al, 2019[53] | Retrospective | Internal | 239 patients with diffuse glioma, 160/79 (training/validation) | SVM | Preoperative T2 | First order statistics, shape, size-based, textural, and wavelet features | Training：  71.3, AUC: 74.1;  Validation：  72.3, AUC:70.2 | Training: 83.5/58.7  validation: 67.9/70.6 |
| Genetic alterations prediction | Calabrese et al, 2020[54] | Retrospective | Internal | 199 GBM patients | Deep CNN and RF | T1, T1c, T2, T2/FLAIR, susceptibility-weighted, DWI, ASL, and high angular resolution diffusion imaging | Shape, first order grayscale, and higher order grayscale features | IDH mutations:  AUC:95;  ATRX mutations:  AUC:97;  chromosome 7/10 aneuploidies:  AUC:93;  CDKN2 family mutations: AUC:85 | IDH mutations:  93/88;  ATRX mutations:  94/92;  chromosome 7/10 aneuploidies:  90/88;  CDKN2 family mutations:  76/86 |
| Classification of molecular characteristics | Kickingereder et al, 2016[55] | Retrospective | Internal | 152 GBMs | Stochastic gradient boosting machine,  RF, and penalized  LR classifiers | T1, T1c, FLAIR, DWI,  dynamic susceptibility-weighted contrast-enhanced imaging, and precontrast susceptibility-weighted imaging | Multiparametric and multiregional information with histogram quantification of tumor volumes, volume ratios, ADCs, CBF, CBV, and intratumoral susceptibility signals | EGFR (LR model with synthetic minority oversampling technique subsampling):  63, AUC:67;  RTK II (a stochastic gradient boosting model  with synthetic minority oversampling  technique subsampling and a RF model without subsampling):  61/61, AUC:62/53 | EGFR (LR model with synthetic minority oversampling technique subsampling): 71/53  RTK II (a stochastic gradient boosting model  with synthetic minority oversampling  technique subsampling): 62/60  RTK II (a RF model without subsampling): 53/69 |
| Tumor cell analysis | | | | | | | | | |
| Tumor cell density predictions | Hu et al, 2019[56] | Prospective | Internal | 82 images from 18 patients with primary GBM | TL | T1c, T2, DTI and dynamic susceptibility contrast | rCBV, T1c, MD and FA | Pearson correlation coeffcients:  88;  mean absolute error:  5.66 | NA |
| Tumor cells classification | Cakmakci et al, 2020[57] | Retrospective | Internal | Glioma and control samples (n = 565) | RF | High resolution magic angle spinning nuclear MRS | Metabolites and ppm signatures | AUC (tumor cells and controls): 85.6;  AUC (benign and malignant samples): 87.1 | NA |
| Brain tumor growth prediction | Pasquini et al, 2020[58] | Retrospective | BraTS 2014 and  Internal | 9 clinical LGG patients and 9 BraTS 2014 dataset HGG patients | GAN | T1, T1c, T2, and FLAIR | DL feature | Jaccard index: 78.97;  Dice coefficient: 88.26 | 89.61/88.78 |
| Characterization of active and infiltrative tumorous subregions from normal tissues | Fathi Kazerooni et al, 2018[59] | Prospective | Internal | Fifty-one tissue specimens from 10 patients | SVM | T1, T1c, T2, T2/FLAIR, T2-relaxometry, DWI, DTI, and dynamic susceptibility  contrast | CBV, FLAIR, MD, and high-resolution T2 image | Normal tissues from infiltrative edema: 93.3, AUC:98.8;  normal tissues from active tumors: 100, AUC:100;  infiltrative edema from active tumors: 96.6, AUC:100 | Normal tissues from infiltrative edema: 94.6/89.8;  normal tissues from active tumors:100/100;  infiltrative edema from active tumors: 85.4/100 |

AI: artificial intelligence; MRI: magnetic resonance imaging; BraTS: Brain Tumor Segmentation; T1: T1-weighted; T2: T2-weighted; T1c: T1-weighted contrast-enhanced; FLAIR: fluid-attenuated inversion recovery; DSC: dice similarity coefficient; GBM: glioblastoma; 3D: three-dimensional; DL: deep learning; NA: not available; Internal: subjects were recruited from insitutional and/or public through media channels; DWI: diffusion weighted imaging; ADC: apparent diffusion coefficient; rCBV: relative cerebral blood volume; AUC: area under the receiver operating characteristic curve; CNN: convolutional neural network; DTI: diffusion tensor imaging; PWI: perfusion weighted imaging; SVM: support vector machine; RF: random forest; ANN: artificial neural network; LGG: low-grade glioma; TCGA: The Cancer Genome Atlas; PCNSL: primary central nervous system lymphoma; LASSO: least absolute shrinkage and selection operator; TICA: The Cancer Imaging Archive; HGG: high-grade glioma; XGBoost: eXtreme Gradient Boosting; MRS: magnetic resonance spectroscopy; 2D: two-dimensional; FA: fractional anisotropy; MD: mean diffusivity; LR: logistic regression; DKI: diffusional kurtosis imaging; ASL: arterial spin labeling; TTP: true tumor progression; GAN: generative adversarial network; OS: overall survival; IDH: isocitrate dehydrogenase; RNN: recurrent neural network; MGMT: methylation of O6-Methylguanine-DNA methyltransferase; TL: transfer learning; ATRX: alpha thalassemia/mental retardation syndrome X-linked; CGGA: Chinese Glioma Genome Atlas; MPRAGE: magnetization prepared rapid gradient echo; TERT: telomerase reverse transcriptase promoter; CBF: cerebral blood flow; TP53: tumor protein 53; VEGF: vascular endothelial growth factor; CDKN: cyclin-dependent kinase inhibitor;

EGFR: epidermal growth factor receptor; RTKII: receptor tyrosine kinase II;

[1] Z. Liu, L. Tong, L. Chen, F. Zhou, Z. Jiang, Q. Zhang, Y. Wang, C. Shan, L. Li, and H. Zhou, CANet: Context Aware Network for Brain Glioma Segmentation. IEEE Trans Med Imaging 40 (2021) 1763-1777.

[2] J. Fu, K. Singhrao, X.S. Qi, Y. Yang, D. Ruan, and J.H. Lewis, Three-dimensional multipath DenseNet for improving automatic segmentation of glioblastoma on pre-operative multimodal MR images. Med Phys 48 (2021) 2859-2866.

[3] A. Khosravanian, M. Rahmanimanesh, P. Keshavarzi, and S. Mozaffari, Fast level set method for glioma brain tumor segmentation based on Superpixel fuzzy clustering and lattice Boltzmann method. Comput Methods Programs Biomed 198 (2021) 105809.

[4] J.E. Park, S. Ham, H.S. Kim, S.Y. Park, J. Yun, H. Lee, S.H. Choi, and N. Kim, Diffusion and perfusion MRI radiomics obtained from deep learning segmentation provides reproducible and comparable diagnostic model to human in post-treatment glioblastoma. Eur Radiol 31 (2021) 3127-3137.

[5] R. Rahmat, K. Saednia, M.R. Haji Hosseini Khani, M. Rahmati, R. Jena, and S.J. Price, Multi-scale segmentation in GBM treatment using diffusion tensor imaging. Comput Biol Med 123 (2020) 103815.

[6] J. Amin, M. Sharif, M. Raza, T. Saba, and M.A. Anjum, Brain tumor detection using statistical and machine learning method. Comput Methods Programs Biomed 177 (2019) 69-79.

[7] A.P. Marcus, H.J. Marcus, S.J. Camp, D. Nandi, N. Kitchen, and L. Thorne, Improved Prediction of Surgical Resectability in Patients with Glioblastoma using an Artificial Neural Network. Sci Rep 10 (2020) 5143.

[8] N. Shusharina, J. Söderberg, D. Edmunds, F. Löfman, H. Shih, and T. Bortfeld, Automated delineation of the clinical target volume using anatomically constrained 3D expansion of the gross tumor volume. Radiother Oncol 146 (2020) 37-43.

[9] J. Basha, N. Bacanin, N. Vukobrat, M. Zivkovic, K. Venkatachalam, S. Hubálovský, and P. Trojovský, Chaotic Harris Hawks Optimization with Quasi-Reflection-Based Learning: An Application to Enhance CNN Design. Sensors (Basel) 21 (2021).

[10] S.A. Abdelaziz Ismael, A. Mohammed, and H. Hefny, An enhanced deep learning approach for brain cancer MRI images classification using residual networks. Artif Intell Med 102 (2020) 101779.

[11] W. Xia, B. Hu, H. Li, W. Shi, Y. Tang, Y. Yu, C. Geng, Q. Wu, L. Yang, Z. Yu, D. Geng, and Y. Li, Deep Learning for Automatic Differential Diagnosis of Primary Central Nervous System Lymphoma and Glioblastoma: Multi-Parametric Magnetic Resonance Imaging Based Convolutional Neural Network Model. J Magn Reson Imaging 54 (2021) 880-887.

[12] J. Yun, J.E. Park, H. Lee, S. Ham, N. Kim, and H.S. Kim, Radiomic features and multilayer perceptron network classifier: a robust MRI classification strategy for distinguishing glioblastoma from primary central nervous system lymphoma. Sci Rep 9 (2019) 5746.

[13] I. Shin, H. Kim, S.S. Ahn, B. Sohn, S. Bae, J.E. Park, H.S. Kim, and S.K. Lee, Development and Validation of a Deep Learning-Based Model to Distinguish Glioblastoma from Solitary Brain Metastasis Using Conventional MR Images. AJNR Am J Neuroradiol 42 (2021) 838-844.

[14] Z. Qian, Y. Li, Y. Wang, L. Li, R. Li, K. Wang, S. Li, K. Tang, C. Zhang, X. Fan, B. Chen, and W. Li, Differentiation of glioblastoma from solitary brain metastases using radiomic machine-learning classifiers. Cancer Lett 451 (2019) 128-135.

[15] Z. Ning, J. Luo, Q. Xiao, L. Cai, Y. Chen, X. Yu, J. Wang, and Y. Zhang, Multi-modal magnetic resonance imaging-based grading analysis for gliomas by integrating radiomics and deep features. Ann Transl Med 9 (2021) 298.

[16] Z.A. Al-Saffar, and T. Yildirim, A hybrid approach based on multiple Eigenvalues selection (MES) for the automated grading of a brain tumor using MRI. Comput Methods Programs Biomed 201 (2021) 105945.

[17] J.L. Quon, W. Bala, L.C. Chen, J. Wright, L.H. Kim, M. Han, K. Shpanskaya, E.H. Lee, E. Tong, M. Iv, J. Seekins, M.P. Lungren, K.R.M. Braun, T.Y. Poussaint, S. Laughlin, M.D. Taylor, R.M. Lober, H. Vogel, P.G. Fisher, G.A. Grant, V. Ramaswamy, N.A. Vitanza, C.Y. Ho, M.S.B. Edwards, S.H. Cheshier, and K.W. Yeom, Deep Learning for Pediatric Posterior Fossa Tumor Detection and Classification: A Multi-Institutional Study. AJNR Am J Neuroradiol 41 (2020) 1718-1725.

[18] H. Luo, Q. Zhuang, Y. Wang, A. Abudumijiti, K. Shi, A. Rominger, H. Chen, Z. Yang, V. Tran, G. Wu, Z. Li, Z. Fan, Z. Qi, Y. Guo, J. Yu, and Z. Shi, A novel image signature-based radiomics method to achieve precise diagnosis and prognostic stratification of gliomas. Lab Invest 101 (2021) 450-462.

[19] N.Q.K. Le, T.N.K. Hung, D.T. Do, L.H.T. Lam, L.H. Dang, and T.-T. Huynh, Radiomics-based machine learning model for efficiently classifying transcriptome subtypes in glioblastoma patients from MRI. Comput Biol Med 132 (2021) 104320.

[20] C.-F. Lu, F.-T. Hsu, K.L.-C. Hsieh, Y.-C.J. Kao, S.-J. Cheng, J.B.-K. Hsu, P.-H. Tsai, R.-J. Chen, C.-C. Huang, Y. Yen, and C.-Y. Chen, Machine Learning-Based Radiomics for Molecular Subtyping of Gliomas. Clin Cancer Res 24 (2018) 4429-4436.

[21] E. Diamandis, C.P.S. Gabriel, U. Würtemberger, K. Guggenberger, H. Urbach, O. Staszewski, S. Lassmann, O. Schnell, J. Grauvogel, I. Mader, and D.H. Heiland, MR-spectroscopic imaging of glial tumors in the spotlight of the 2016 WHO classification. J Neurooncol 139 (2018) 431-440.

[22] Y. Zhuge, H. Ning, P. Mathen, J.Y. Cheng, A.V. Krauze, K. Camphausen, and R.W. Miller, Automated glioma grading on conventional MRI images using deep convolutional neural networks. Med Phys 47 (2020) 3044-3053.

[23] Z. Zhang, J. Xiao, S. Wu, F. Lv, J. Gong, L. Jiang, R. Yu, and T. Luo, Deep Convolutional Radiomic Features on Diffusion Tensor Images for Classification of Glioma Grades. J Digit Imaging 33 (2020) 826-837.

[24] S. Takahashi, W. Takahashi, S. Tanaka, A. Haga, T. Nakamoto, Y. Suzuki, A. Mukasa, S. Takayanagi, Y. Kitagawa, T. Hana, T. Nejo, M. Nomura, K. Nakagawa, and N. Saito, Radiomics Analysis for Glioma Malignancy Evaluation Using Diffusion Kurtosis and Tensor Imaging. Int J Radiat Oncol Biol Phys 105 (2019) 784-791.

[25] Q. Tian, L.-F. Yan, X. Zhang, X. Zhang, Y.-C. Hu, Y. Han, Z.-C. Liu, H.-Y. Nan, Q. Sun, Y.-Z. Sun, Y. Yang, Y. Yu, J. Zhang, B. Hu, G. Xiao, P. Chen, S. Tian, J. Xu, W. Wang, and G.-B. Cui, Radiomics strategy for glioma grading using texture features from multiparametric MRI. J Magn Reson Imaging 48 (2018) 1518-1528.

[26] K.S. Choi, S.-H. You, Y. Han, J.C. Ye, B. Jeong, and S.H. Choi, Improving the Reliability of Pharmacokinetic Parameters at Dynamic Contrast-enhanced MRI in Astrocytomas: A Deep Learning Approach. Radiology 297 (2020) 178-188.

[27] C. Qi, Y. Li, X. Fan, Y. Jiang, R. Wang, S. Yang, L. Meng, T. Jiang, and S. Li, A quantitative SVM approach potentially improves the accuracy of magnetic resonance spectroscopy in the preoperative evaluation of the grades of diffuse gliomas. Neuroimage Clin 23 (2019) 101835.

[28] H. Akbari, S. Rathore, S. Bakas, M.P. Nasrallah, G. Shukla, E. Mamourian, M. Rozycki, S.J. Bagley, J.D. Rudie, A.E. Flanders, A.P. Dicker, A.S. Desai, D.M. O'Rourke, S. Brem, R. Lustig, S. Mohan, R.L. Wolf, M. Bilello, M. Martinez-Lage, and C. Davatzikos, Histopathology-validated machine learning radiographic biomarker for noninvasive discrimination between true progression and pseudo-progression in glioblastoma. Cancer 126 (2020) 2625-2636.

[29] N. Elshafeey, A. Kotrotsou, A. Hassan, N. Elshafei, I. Hassan, S. Ahmed, S. Abrol, A. Agarwal, K. El Salek, S. Bergamaschi, J. Acharya, F.E. Moron, M. Law, G.N. Fuller, J.T. Huse, P.O. Zinn, and R.R. Colen, Multicenter study demonstrates radiomic features derived from magnetic resonance perfusion images identify pseudoprogression in glioblastoma. Nat Commun 10 (2019) 3170.

[30] M. Li, H. Tang, M.D. Chan, X. Zhou, and X. Qian, DC-AL GAN: Pseudoprogression and true tumor progression of glioblastoma multiform image classification based on DCGAN and AlexNet. Med Phys 47 (2020) 1139-1150.

[31] M. Ismail, V. Hill, V. Statsevych, R. Huang, P. Prasanna, R. Correa, G. Singh, K. Bera, N. Beig, R. Thawani, A. Madabhushi, M. Aahluwalia, and P. Tiwari, Shape Features of the Lesion Habitat to Differentiate Brain Tumor Progression from Pseudoprogression on Routine Multiparametric MRI: A Multisite Study. AJNR Am J Neuroradiol 39 (2018) 2187-2193.

[32] Z.-Q. Pan, S.-J. Zhang, X.-L. Wang, Y.-X. Jiao, and J.-J. Qiu, Machine Learning Based on a Multiparametric and Multiregional Radiomics Signature Predicts Radiotherapeutic Response in Patients with Glioblastoma. Behav Neurol 2020 (2020) 1712604.

[33] X. Zhang, D. Lu, P. Gao, Q. Tian, H. Lu, X. Xu, X. He, and Y. Liu, Survival-relevant high-risk subregion identification for glioblastoma patients: the MRI-based multiple instance learning approach. Eur Radiol 30 (2020) 5602-5610.

[34] M. Soltani, A. Bonakdar, N. Shakourifar, R. Babaie, and K. Raahemifar, Efficacy of Location-Based Features for Survival Prediction of Patients With Glioblastoma Depending on Resection Status. Front Oncol 11 (2021) 661123.

[35] Y. Chang, K. Lafata, W. Sun, C. Wang, Z. Chang, J.P. Kirkpatrick, and F.-F. Yin, An investigation of machine learning methods in delta-radiomics feature analysis. PLoS One 14 (2019) e0226348.

[36] P. Sanghani, B.T. Ang, N.K.K. King, and H. Ren, Overall survival prediction in glioblastoma multiforme patients from volumetric, shape and texture features using machine learning. Surg Oncol 27 (2018) 709-714.

[37] K.E. Emblem, M.C. Pinho, F.G. Zöllner, P. Due-Tonnessen, J.K. Hald, L.R. Schad, T.R. Meling, O. Rapalino, and A. Bjornerud, A generic support vector machine model for preoperative glioma survival associations. Radiology 275 (2015) 228-234.

[38] K.S. Choi, S.H. Choi, and B. Jeong, Prediction of IDH genotype in gliomas with dynamic susceptibility contrast perfusion MR imaging using an explainable recurrent neural network. Neuro Oncol 21 (2019) 1197-1209.

[39] Y. Tan, S.-T. Zhang, J.-W. Wei, D. Dong, X.-C. Wang, G.-Q. Yang, J. Tian, and H. Zhang, A radiomics nomogram may improve the prediction of IDH genotype for astrocytoma before surgery. Eur Radiol 29 (2019) 3325-3337.

[40] K. Chang, H.X. Bai, H. Zhou, C. Su, W.L. Bi, E. Agbodza, V.K. Kavouridis, J.T. Senders, A. Boaro, A. Beers, B. Zhang, A. Capellini, W. Liao, Q. Shen, X. Li, B. Xiao, J. Cryan, S. Ramkissoon, L. Ramkissoon, K. Ligon, P.Y. Wen, R.S. Bindra, J. Woo, O. Arnaout, E.R. Gerstner, P.J. Zhang, B.R. Rosen, L. Yang, R.Y. Huang, and J. Kalpathy-Cramer, Residual Convolutional Neural Network for the Determination of Status in Low- and High-Grade Gliomas from MR Imaging. Clin Cancer Res 24 (2018) 1073-1081.

[41] S. Bisdas, H. Shen, S. Thust, V. Katsaros, G. Stranjalis, C. Boskos, S. Brandner, and J. Zhang, Texture analysis- and support vector machine-assisted diffusional kurtosis imaging may allow in vivo gliomas grading and IDH-mutation status prediction: a preliminary study. Sci Rep 8 (2018) 6108.

[42] P. Eichinger, E. Alberts, C. Delbridge, S. Trebeschi, A. Valentinitsch, S. Bette, T. Huber, J. Gempt, B. Meyer, J. Schlegel, C. Zimmer, J.S. Kirschke, B.H. Menze, and B. Wiestler, Diffusion tensor image features predict IDH genotype in newly diagnosed WHO grade II/III gliomas. Sci Rep 7 (2017) 13396.

[43] C.G.B. Yogananda, B.R. Shah, S.S. Nalawade, G.K. Murugesan, F.F. Yu, M.C. Pinho, B.C. Wagner, B. Mickey, T.R. Patel, B. Fei, A.J. Madhuranthakam, and J.A. Maldjian, MRI-Based Deep-Learning Method for Determining Glioma Promoter Methylation Status. AJNR Am J Neuroradiol 42 (2021) 845-852.

[44] S. Zhang, H. Sun, X. Su, X. Yang, W. Wang, X. Wan, Q. Tan, N. Chen, Q. Yue, and Q. Gong, Automated machine learning to predict the co-occurrence of isocitrate dehydrogenase mutations and O -methylguanine-DNA methyltransferase promoter methylation in patients with gliomas. J Magn Reson Imaging 54 (2021) 197-205.

[45] Y. Li, X. Liu, Z. Qian, Z. Sun, K. Xu, K. Wang, X. Fan, Z. Zhang, S. Li, Y. Wang, and T. Jiang, Genotype prediction of ATRX mutation in lower-grade gliomas using an MRI radiomics signature. Eur Radiol 28 (2018) 2960-2968.

[46] Y. Ren, X. Zhang, W. Rui, H. Pang, T. Qiu, J. Wang, Q. Xie, T. Jin, H. Zhang, H. Chen, Y. Zhang, H. Lu, Z. Yao, J. Zhang, and X. Feng, Noninvasive Prediction of IDH1 Mutation and ATRX Expression Loss in Low-Grade Gliomas Using Multiparametric MR Radiomic Features. J Magn Reson Imaging 49 (2019) 808-817.

[47] X. Su, N. Chen, H. Sun, Y. Liu, X. Yang, W. Wang, S. Zhang, Q. Tan, J. Su, Q. Gong, and Q. Yue, Automated machine learning based on radiomics features predicts H3 K27M mutation in midline gliomas of the brain. Neuro Oncol 22 (2020) 393-401.

[48] J. Liu, F. Chen, C. Pan, M. Zhu, X. Zhang, L. Zhang, and H. Liao, A Cascaded Deep Convolutional Neural Network for Joint Segmentation and Genotype Prediction of Brainstem Gliomas. IEEE Trans Biomed Eng 65 (2018) 1943-1952.

[49] R. Fukuma, T. Yanagisawa, M. Kinoshita, T. Shinozaki, H. Arita, A. Kawaguchi, M. Takahashi, Y. Narita, Y. Terakawa, N. Tsuyuguchi, Y. Okita, M. Nonaka, S. Moriuchi, M. Takagaki, Y. Fujimoto, J. Fukai, S. Izumoto, K. Ishibashi, Y. Nakajima, T. Shofuda, D. Kanematsu, E. Yoshioka, Y. Kodama, M. Mano, K. Mori, K. Ichimura, Y. Kanemura, and H. Kishima, Prediction of IDH and TERT promoter mutations in low-grade glioma from magnetic resonance images using a convolutional neural network. Sci Rep 9 (2019) 20311.

[50] Z. Akkus, I. Ali, J. Sedlář, J.P. Agrawal, I.F. Parney, C. Giannini, and B.J. Erickson, Predicting Deletion of Chromosomal Arms 1p/19q in Low-Grade Gliomas from MR Images Using Machine Intelligence. J Digit Imaging 30 (2017) 469-476.

[51] E.D.H. Gates, J.S. Lin, J.S. Weinberg, J. Hamilton, S.S. Prabhu, J.D. Hazle, G.N. Fuller, V. Baladandayuthapani, D. Fuentes, and D. Schellingerhout, Guiding the first biopsy in glioma patients using estimated Ki-67 maps derived from MRI: conventional versus advanced imaging. Neuro Oncol 21 (2019) 527-536.

[52] Y. Li, Z. Qian, K. Xu, K. Wang, X. Fan, S. Li, T. Jiang, X. Liu, and Y. Wang, MRI features predict p53 status in lower-grade gliomas via a machine-learning approach. Neuroimage Clin 17 (2018) 306-311.

[53] Z. Sun, Y. Li, Y. Wang, X. Fan, K. Xu, K. Wang, S. Li, Z. Zhang, T. Jiang, and X. Liu, Radiogenomic analysis of vascular endothelial growth factor in patients with diffuse gliomas. Cancer Imaging 19 (2019) 68.

[54] E. Calabrese, J.E. Villanueva-Meyer, and S. Cha, A fully automated artificial intelligence method for non-invasive, imaging-based identification of genetic alterations in glioblastomas. Sci Rep 10 (2020) 11852.

[55] P. Kickingereder, D. Bonekamp, M. Nowosielski, A. Kratz, M. Sill, S. Burth, A. Wick, O. Eidel, H.-P. Schlemmer, A. Radbruch, J. Debus, C. Herold-Mende, A. Unterberg, D. Jones, S. Pfister, W. Wick, A. von Deimling, M. Bendszus, and D. Capper, Radiogenomics of Glioblastoma: Machine Learning-based Classification of Molecular Characteristics by Using Multiparametric and Multiregional MR Imaging Features. Radiology 281 (2016) 907-918.

[56] L.S. Hu, H. Yoon, J.M. Eschbacher, L.C. Baxter, A.C. Dueck, A. Nespodzany, K.A. Smith, P. Nakaji, Y. Xu, L. Wang, J.P. Karis, A.J. Hawkins-Daarud, K.W. Singleton, P.R. Jackson, B.J. Anderies, B.R. Bendok, R.S. Zimmerman, C. Quarles, A.B. Porter-Umphrey, M.M. Mrugala, A. Sharma, J.M. Hoxworth, M.G. Sattur, N. Sanai, P.E. Koulemberis, C. Krishna, J.R. Mitchell, T. Wu, N.L. Tran, K.R. Swanson, and J. Li, Accurate Patient-Specific Machine Learning Models of Glioblastoma Invasion Using Transfer Learning. AJNR Am J Neuroradiol 40 (2019) 418-425.

[57] D. Cakmakci, E.O. Karakaslar, E. Ruhland, M.-P. Chenard, F. Proust, M. Piotto, I.J. Namer, and A.E. Cicek, Machine learning assisted intraoperative assessment of brain tumor margins using HRMAS NMR spectroscopy. PLoS Comput Biol 16 (2020) e1008184.

[58] A. Elazab, C. Wang, S.J.S. Gardezi, H. Bai, Q. Hu, T. Wang, C. Chang, and B. Lei, GP-GAN: Brain tumor growth prediction using stacked 3D generative adversarial networks from longitudinal MR Images. Neural Netw 132 (2020) 321-332.

[59] A. Fathi Kazerooni, M. Nabil, M. Zeinali Zadeh, K. Firouznia, F. Azmoudeh-Ardalan, A.F. Frangi, C. Davatzikos, and H. Saligheh Rad, Characterization of active and infiltrative tumorous subregions from normal tissue in brain gliomas using multiparametric MRI. J Magn Reson Imaging 48 (2018) 938-950.
